# Crystallographic and Computational Insights into Isoform-Selective Dynamics in Nitric Oxide Synthase

**DOI:** 10.1021/acs.biochem.3c00601

**Published:** 2024-02-28

**Authors:** Huiying Li, Christine D. Hardy, Cory T. Reidl, Qing Jing, Fengtian Xue, Maris Cinelli, Richard B. Silverman, Thomas L. Poulos

**Affiliations:** † Departments of Molecular Biology and Biochemistry, Pharmaceutical Sciences, and Chemistry, University of California, Irvine, California 92697-3900, United States; ‡ Department of Chemistry, Department of Molecular Biosciences, Chemistry of Life Processes Institute, Center for Developmental Therapeutics, Northwestern University, 2145 Sheridan Road, Evanston, Illinois 60208-3113, United States; § Department of Pharmacology, Feinberg School of Medicine, Northwestern University, Chicago, Illinois 60611, United States

## Abstract

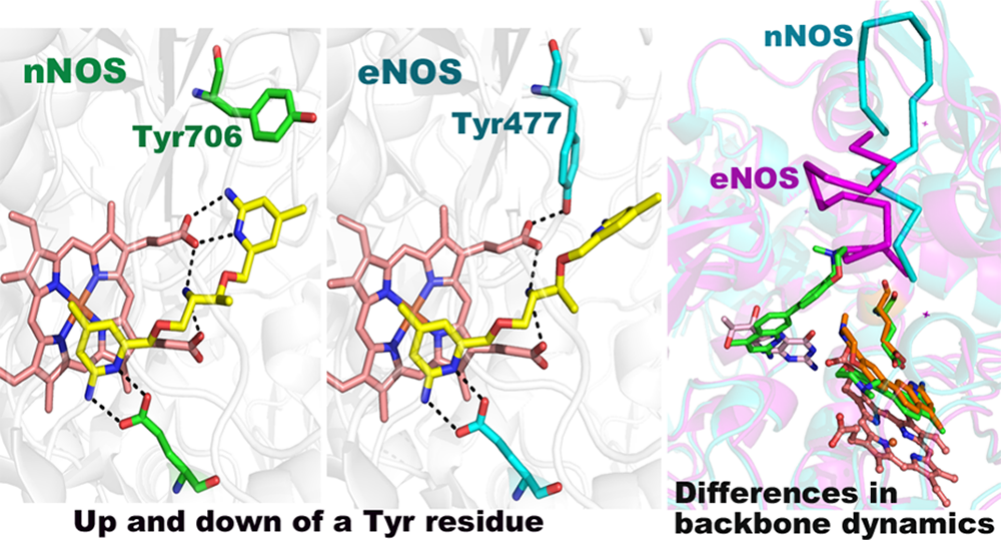

In our efforts to develop inhibitors selective for neuronal nitric oxide synthase (nNOS) over endothelial nitric oxide synthase (eNOS), we found that nNOS can undergo conformational changes in response to inhibitor binding that does not readily occur in eNOS. One change involves movement of a conserved tyrosine, which hydrogen bonds to one of the heme propionates, but in the presence of an inhibitor, changes conformation, enabling part of the inhibitor to hydrogen bond with the heme propionate. This movement does not occur as readily in eNOS and may account for the reason why these inhibitors bind more tightly to nNOS. A second structural change occurs upon the binding of a second inhibitor molecule to nNOS, displacing the pterin cofactor. Binding of this second site inhibitor requires structural changes at the dimer interface, which also occurs more readily in nNOS than in eNOS. Here, we used a combination of crystallography, mutagenesis, and computational methods to better understand the structural basis for these differences in NOS inhibitor binding. Computational results show that a conserved tyrosine near the primary inhibitor binding site is anchored more tightly in eNOS than in nNOS, allowing for less flexibility of this residue. We also find that the inefficiency of eNOS to bind a second inhibitor molecule is likely due to the tighter dimer interface in eNOS compared with nNOS. This study provides a better understanding of how subtle structural differences in NOS isoforms can result in substantial dynamic differences that can be exploited in the development of isoform-selective inhibitors.

## Introduction

Nitric oxide (NO) is an important signaling molecule critical in the cardiovascular, neuronal, and immune systems.^1−3^ The enzyme responsible for generating NO is nitric oxide synthase (NOS), a P450-like heme thiolate enzyme that oxidizes l-arginine to NO and L-citrulline. Humans and other mammals express three different NOS isoforms: endothelial NOS (eNOS), neuronal NOS (nNOS), and inducible NOS (iNOS). nNOS is an important therapeutic target since the overproduction of NO by nNOS in the brain is associated with a number of neurodegenerative diseases.^4−6^ Thus, nNOS is an attractive target for therapeutic intervention.

**Figure 1:**
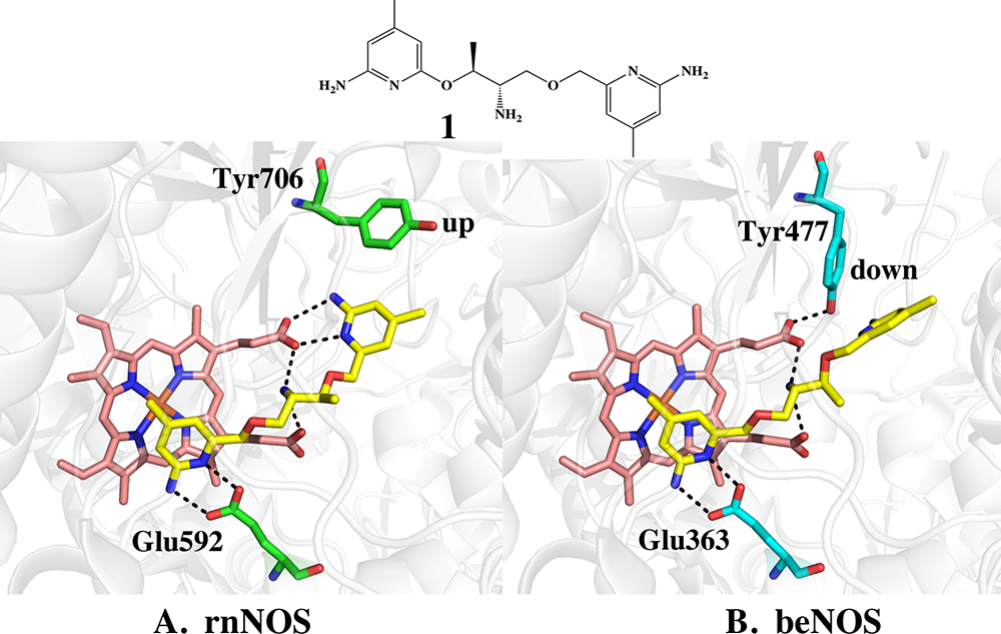
Crystal structure of **1** bound to (A) rnNOS (4k5g) and (B) beNOS (4k5k). In rnNOS Tyr706 rotates to the “up” position which enables the tail aminopyridine of **1** extending out of the active site to H-bond with a heme propionate. In beNOS bound to **1**, Tyr477 remains in the “down” position which prevents the inhibitor from H-bonding with the heme propionate. The electron density for the tail aminopyridine extending out of the active in beNOS is also quite weak,^9^ indicating that this group is partially disordered.

Selectivity for nNOS over eNOS is critically important since inhibiting eNOS can seriously compromise proper cardiovascular function. Our laboratories have employed a combination of chemistry, computer modeling, and crystallography which has resulted in the development of inhibitors that are up to 4,000-fold more selective for nNOS over eNOS.^7,8^ These studies have resulted in a large number of crystal structures, some of which illustrate the heretofore unexplained dynamic differences between nNOS and eNOS. Figure 1 provides a first example. In compound **1**,^9^ one aminopyridine penetrates the active site over the heme where it can establish H-bonds with the invariant active site Glu. In rat nNOS (rnNOS), the tail end aminopyridine extends out of the active site, where it establishes H-bonds with a heme propionate. For the inhibitor to interact with the heme propionate, Tyr706 must rotate out of the way (Figure 1A). This change was not observed in inhibitor-bound bovine eNOS (beNOS), where Tyr477 remains in place (Figure 1B). This difference may account, in part, for the 96-fold greater affinity of **1** for rnNOS or beNOS.^9^ This difference in Tyr mobility also has been observed with several other inhibitors.^10^

**Figure 2:**
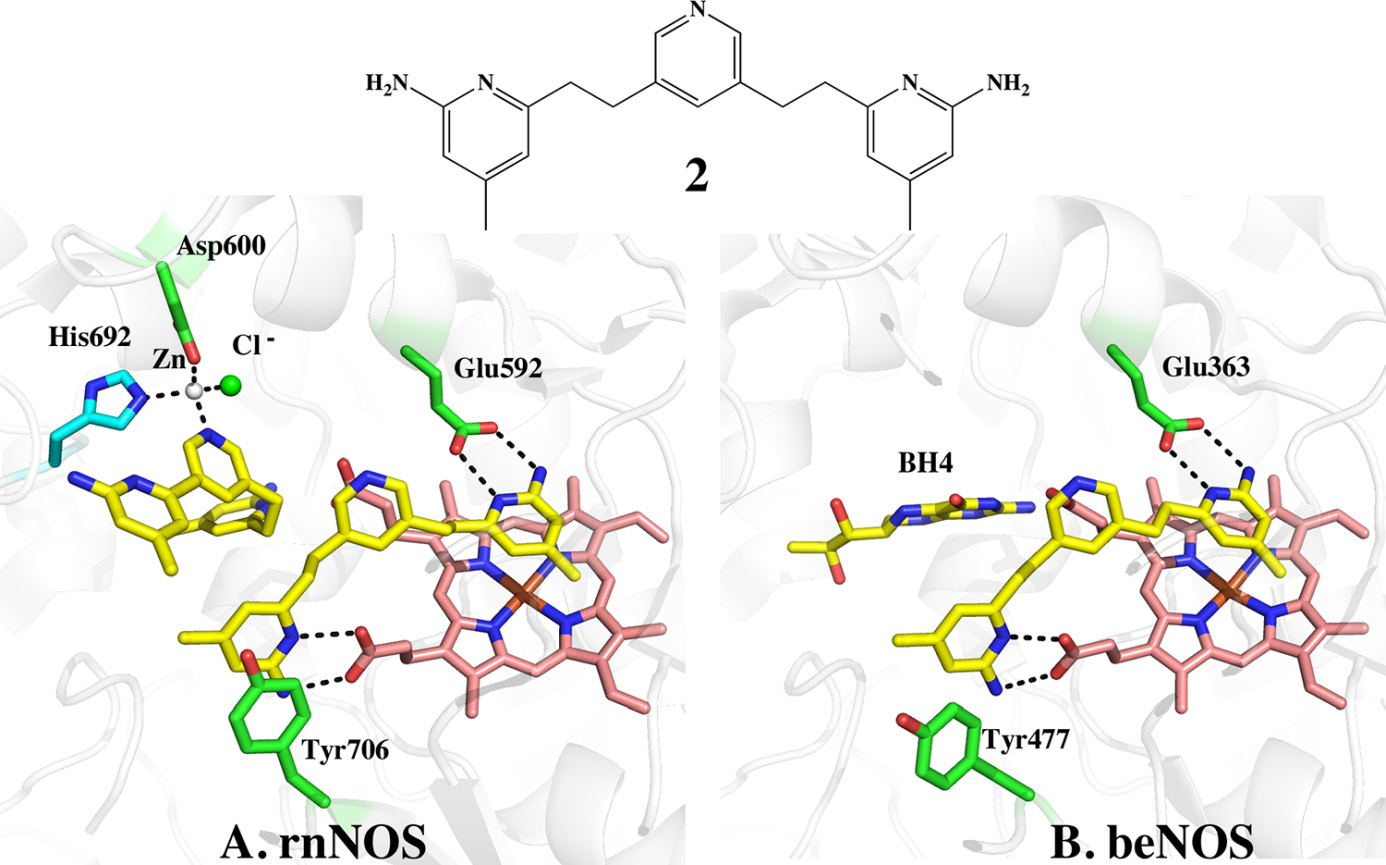
Crystal structure of **2** bound to (A) rnNOS (3n5w) and (B) beNOS (3n5t). In rnNOS, a second molecule of **2** displaces BH_4_ which enables one of the aminopyridines to H-bond with a heme propionate. This places the central pyridine of the second **2** molecule in a position to form part of a tetrahedral coordination sphere around a Zn^2+^ cation. The Zn^2+^ is near the interface of the nNOS dimer and His692 from molecule B and Asp600 from molecule A form part of the Zn^2+^ coordination sphere. In beNOS, only one molecule of **2** binds in the active site, and no changes are observed in the BH_4_ binding pocket.

A second significant difference is that for some inhibitors, two inhibitor molecules bind to nNOS. One binds in the active site, as expected, and the second binds in the cofactor pterin (BH_4_) pocket. The first example where this was observed^11,12^ is shown in Figure 2. In both rnNOS and beNOS, **2** binds in the active site with one aminopyridine forming H-bonds with the active site Glu. However, in rnNOS, a second molecule of **2** binds with one aminopyridine in the cofactor BH_4_ pocket. The central linker pyridine is then in a position to participate in the tetrahedral coordination of a zinc ion (Figure 2A). A chloride ion completes the tetrahedral coordination of the zinc. Zinc was not included in any of the buffers so we assume the affinity must be quite high. Since we have not observed zinc binding in structures without a second inhibitor bound, the second inhibitor pyridine nitrogen must be essential for zinc binding in rnNOS. In contrast, in beNOS BH_4_ remains intact and zinc binding is not observed (Figure 2B).

**Figure 3:**
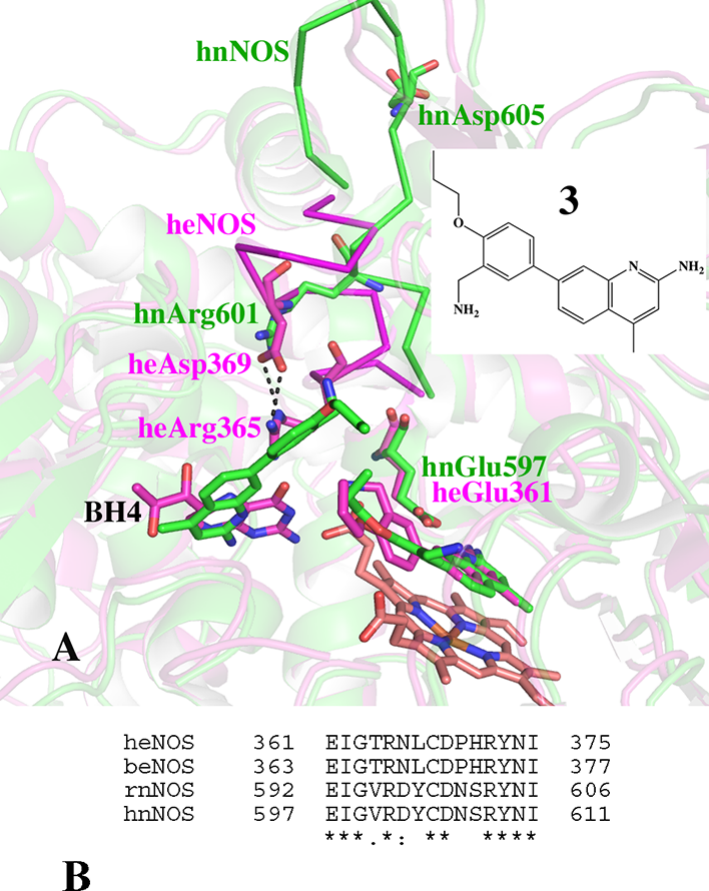
(A) Overlaid crystal structures of **3** bound to hnNOS (6png, green) and heNOS (6poy, magenta). In hnNOS, two molecules of **3** bind, one as expected in the active site, and the other displacing BH_4_. To make room for the second **3** molecule, a substantial local rearrangement in the structure is required in the region highlighted by the green (hnNOS, residues 598–611) and magenta (heNOS, residues 362–375) ribbons. In heNOS, only one **3** molecule binds in the active site. (B) Sequence alignment of the backbone region that undergoes rearrangement upon the inhibitor binding in nNOS but not in eNOS.

More recently, we have observed that the binding of the second inhibitor to the BH_4_ site in nNOS can sometimes be accompanied by a significant backbone structural change in nNOS that does not occur in eNOS. Inhibitor **3** (Figure 3) provides one example.^13^ In both human NOS (hnNOS) and human eNOS (heNOS), **3** binds in the active site, as expected. However, in hnNOS, a second **3** molecule displaces BH_4_ which enables the aminoquinoline to establish H-bonds with the heme propionate. To accommodate the bulky **3** in the BH_4_ site, the nearby salt bridge, Arg601 and Asp605, is pushed away resulting in the collapse of the key helix where the active site Glu597 resides into a random loop (green loop in Figure 3). In heNOS, since **3** does not displace BH_4_, no structural changes were observed in the protein backbone. The salt bridge Arg365/Asp369 in heNOS (equivalent to salt bridge Arg601/Asp605 in hnNOS) remains intact, as shown in Figure 3 as part of the magenta ribbon.

To better understand the significance of the observed differences in structural changes upon inhibitor binding between NOS isoforms, we used a combination of computational methods and crystal structures to further probe these isoform-selective dynamics.

## Methods

### Synthetic Chemistry

**Figure 4:**
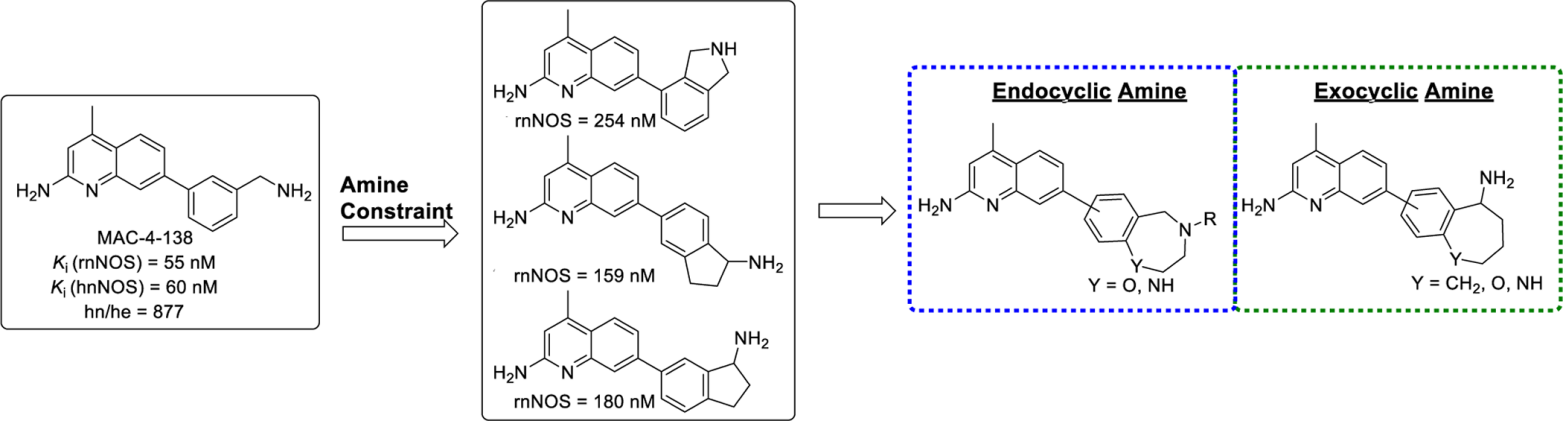
Structural inspiration for this series.

Figure 4 outlines the rationale for the new inhibitors described in this study.

**Scheme 1:**
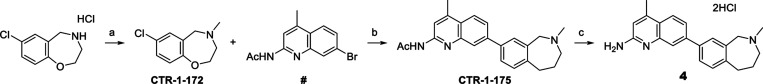
Preparation of Aminoquinoline **4** Reagents and conditions: (a) paraformaldehyde, NaBH_3_CN, AcOH, r.t., 5 h; (b) (i) **CTR-1–172**, B_2_(OH)_2_, KOAc, XPhos-Pd-G3 (1 mol %), XPhos (2 mol %), EtOH, 80 °C, 2 h; (ii) **##**, K_2_CO_3_, H_2_O, 80 °C, 15 h; (c) (i) K_2_CO_3_, MeOH, reflux; (ii) HCl–MeOH, 15 h.

**Scheme 2:**
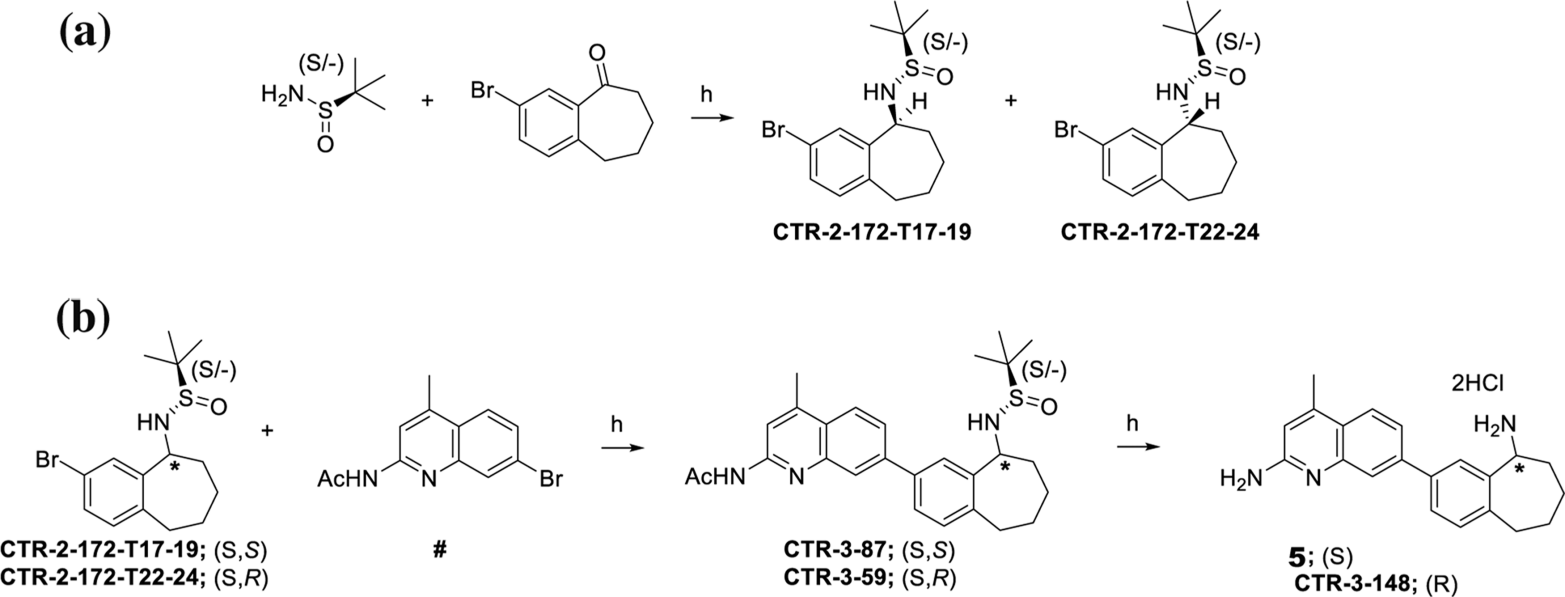
Preparation of Aminoquinolines **5** and **CTR-3-148** Reagents and conditions: (a) (i) THF, titanium(IV) isopropoxide, μwave at 100 °C, 4h, then r.t. 15 h; (ii) NaBH_4_, r.t. 2 h; (b) (i) **CTR-2-172-T17–19** or **CTR-172-T22–24**, B_2_(OH)_2_, KOAc, XPhos-Pd-G3 (1 mol %), XPhos (2 mol %), EtOH, 80 °C, 2 h; (ii) **##**, K_2_CO_3_, H_2_O, 80 °C, 15 h; (h) HCl–MeOH, μwave at 75 °C, 2 × 60 min.

The syntheses of **4** and CTR-3–48 are outlined in Schemes 1 and 2, respectively.

### Computational Methods

MD simulations were carried out with the GPU-optimized pmemd.cuda^14^ in Amber 20. Small molecule ligand parameters were assigned using the GAFF force field^15^ and AM1-BCC charge scheme^15,16^ as implemented in the antechamber module in Amber. Heme parameters were taken from Shahrokh et al.^17^ The starting structures were the highest resolution available which for nNOS was the 1.75 Å rat nNOS structure complexed with the substrate, l-arginine (1OM4), while for eNOS we used the 1.90 Å structure complex with bovine eNOS complexed with ethylisothiourea (1NSE). Another consideration was to use starting structures with the smallest ligands since our goal was to carry out simulations with substrate and ligand-free structures, and those with the smallest ligands come closest to matching ligand-free structures. Although these are the best structures available that meet these criteria, there are short segments that are disordered. However, in a number of other structures, these regions are well-ordered, and we used these to model the disordered regions of the structures used for the simulations. The two structures used that have ordered regions were 6NGL for eNOS and 5VV8 for nNOS. Since the main goal was to test the flexibility of each isoform prior to any ligand binding, the active site ligand was removed. The crystal structures, including crystallographic water molecules, were immersed in an octahedral box of water with a 10 Å cushion and neutralized with Na^+^ ions. Structures were conjugate gradient minimized for 1000 cycles allowing only H atoms and solvent molecules to move followed by 10,000 cycles with no restraints. Equilibration of the minimized structure was carried out in three steps. First, a short 20 ps constant volume MD run was carried out where only H atoms and solvent molecules were allowed to move. Second, a 100 ps constant pressure run was performed in which backbone atoms were restrained by 10 kcal/Å^2^. Third, a 200 ps constant pressure run was performed with no restraints. The final equilibrated structure was used as input for two separate 200 ns runs where each run used a different starting velocity. Since beNOS and rnNOS are dimers, the two runs provided snapshots for two active sites per isoform.

### Construction of Expression Constructs for hnNOS R354A/G357D/E597Q, hnNOS R354A/G357D/N606P, heNOS E361Q, and heNOS P370N Mutants

The heme domain of hnNOS (residues Cys302 to Lys722) was cloned into the NdeI and XhoI sites of pET22b following PCR amplification of the region from pCWori-hnNOS ND (ND, N-terminal domain, which includes both PDZ and heme domains and also contains the R354A and G356D changes).^18,19^ The reverse primer used for cloning of the hnNOS heme domain was designed to introduce a thrombin-cleavage site to encode a thrombin-cleavable C-terminal His6 tag in the expressed protein. The E597Q mutation was introduced into this construct using PrimeStar Max polymerase (Takara) with a standard two-primer site-directed mutagenesis protocol. The hnNOS N606P mutation was introduced using the same site-directed mutagenesis method into a different expression construct, in which the heme domain of hnNOS (residues Cys302 to Lys722) was cloned into pET22b with a forward primer that introduced a thrombin-cleavable N-terminal His6 tag.

The heNOS E361Q mutation was introduced into the full-length pCWori-heNOS expression construct^18^ using site-directed mutagenesis as above. For the heNOS P370N mutant, an expression construct was used in which the heNOS heme domain (Lys67-Trp480) was cloned into the NdeI and XhoI sites of pET22b using a forward primer that introduced a thrombin-cleavable N-terminal His6 tag. The P370N mutation was introduced into this construct as above.

### Protein Expression, Purification, and Crystal Preparation

The protein expression and purification of hnNOS and heNOS^18^ were described in detail previously.^18,20^ The same protocols were followed for hnNOS mutants, R354A/G357D/E597Q and R354A/G357D/N606P, and heNOS mutants E361Q and P370N.

The sitting drop vapor diffusion method was used to grow crystals at 4 °C for the heme domains of hnNOS R354A/G357D, R354A/G357D/E597Q, and R354A/G357D/N606P mutants at a concentration of 7 mg/mL; heNOS WT, E361Q, and P370N mutants were at concentration of 10 mg/mL. The crystal growth conditions were as described previously.^18^ To improve the size and quality of crystals, streak seeding techniques were used at a reduced protein concentration. After extensive crystallization screening, we were unable to obtain diffraction-quality crystals of the hnNOS R354A/G357D/N606P mutant.

For hnNOS, the crystal space group symmetry varied depending on how the heme domain sample was produced. Originally, the hnNOS heme domain was generated by trypsin digest from the full-length protein, which crystallized in space group *P*2_1_2_1_2_1_.^21^ Recently, we cloned the hnNOS heme domain into a pET22b vector.^20^ Although the crystal growth conditions were not changed, the crystals were formed in space group *P*2_1_. All the heNOS crystals for this work were in the *P*2_1_ space group rather than orthorhombic *P*2_1_2_1_2_1_ reported previously^18^ under the same crystal growth conditions.

### X-ray Diffraction Data Collection, Data Processing, and Structural Refinements

The cryogenic (100 K) X-ray diffraction data were collected remotely at the Stanford Synchrotron Radiation Lightsource (SSRL) or the Advanced Light Source (ALS) through data collection control software and a crystal-mounting robot. Raw data frames were indexed, integrated, and scaled using XDS^22^ and scaled with Aimless.^23^ The binding of inhibitors was detected by initial difference Fourier maps calculated with REFMAC.^24^ The inhibitor molecules were then modeled in Coot^25^ and refined using REFMAC and then PHENIX.^26^ Disordering in portions of inhibitors bound in the NOS active sites was often observed, sometimes resulting in poor density quality. However, partial structural features were usually still visible if the contour level of the sigma A weighted 2*m*|*F*_o_| – *D*|*F*_c_| map was dropped to 0.5σ, which afforded the building of reasonable models into the disordered regions. Water molecules were added in PHENIX and checked visually in Coot. The TLS^27^ protocol was implemented in the PHENIX refinements with each subunit as one TLS group. The refined structures were validated through the validation facility in wwPDB before final deposition to the Protein Data Bank. The crystallographic data collection and refinement statistics are reported in Table S1, which includes the PDB codes for all new structures in this study.

## Results and Discussion

### Dynamics of Key Tyr residues, Tyr706 and Tyr477, near the Active Site

Several rnNOS crystal structures have shown that Tyr706, near the active site, normally H-bonded to the heme propionate, can rotate into an “up” conformation (Figure 1), allowing an inhibitor aminopyridine group to replace the Tyr and establish H-bonds with the heme propionate. This conformation was observed more often in inhibitor-bound nNOS structures than in inhibitor-bound eNOS structures, indicating that this Tyr residue may be more mobile in rnNOS.

**Figure 5:**
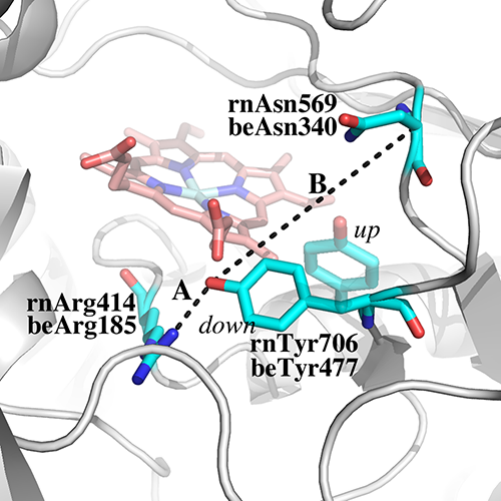
Distances A and B were monitored in MD and SMD runs. In rotating from the “down” to the “up” position, the distance between the Tyr OH group and the Cα atom of the Asn decreases by 7 Å. In the SMD runs, the Tyr OH was “pulled” toward the Asn Cα at 7 Å/ns for a total of 1 ns. For conventional MD runs, distance A was monitored.

To further understand these observations, steered molecular dynamics (SMD) was used to determine the force required to move the Tyr residue H-bonded to the heme propionate (Figure 5) from the “down” to the “up” position. In inhibitor-bound rnNOS crystal structures, the distance between the Tyr side chain OH oxygen and the Cα of a neighboring Asn (distance B in Figure 5) decreases by ∼7 Å. SMD was used to “pull” the Tyr OH toward the Asn Cα by 7 Å while restraining the Cα atoms of both the Tyr and Asn. The two main variables in this calculation are the rate of movement and the force of the pull. A velocity of 7.0 Å/ns^28^ was chosen, and several pulling forces were tested.^29^ As others have found, the total energy required to complete the rotation of the Tyr was relatively insensitive to the choice of force.^30^ The minimum force that gave a smooth linear movement was 10 kcal mol^–1^ Å^–2^. The system was prepared as in a standard MD run except after heating and equilibration a 20 ns *NVT* production run was carried out. One hundred snapshots over the last 10 ns of the production run were used for 1.0 ns SMD runs with a pulling velocity of 7 Å/ns resulting in a 7 Å change in distance. This was repeated 100 times and the Jarzynski average was calculated over the 100 SMD runs^31^ to obtain the potential of mean force (PMF). Since NOS is a dimer, the SMD run was repeated for both active sites in rnNOS and beNOS.

**Figure 6:**
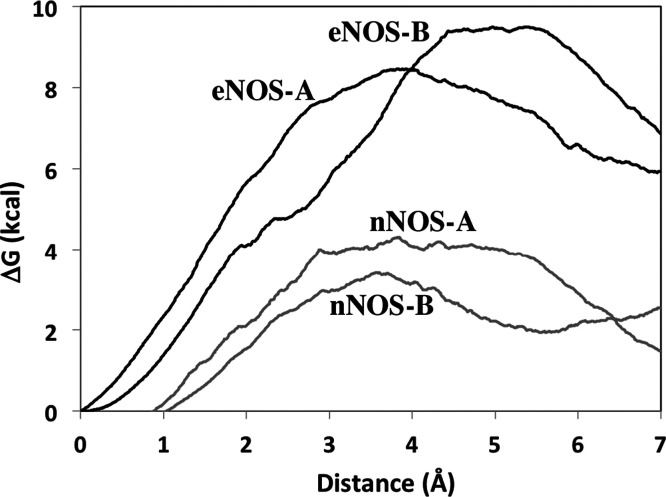
Comparison of the potential of mean force required to rotate the active site Tyr from the “down” to the “up” position in rnNOS and beNOS in both active sites (denoted as NOS-A and NOS-B) of the dimer. Approximately 4–6 kcal/mol more force is required in beNOS as compared to that in rnNOS to complete this movement.

In both rnNOS and beNOS, the Tyr in question H-bonds with both the heme propionate and a neighboring Arg (Figure 5). The distance between the Arg and the Tyr side chain OH was monitored from 200 ns simulations and snapshots saved every 20 ps, giving a total of 10,000 snapshots. Since we ran two 200 ns simulations and monitored the distance in both active sites of the dimer, the Tyr-Arg distance was monitored a total of 40,000 times. In beNOS, the Tyr-Arg distance remained ≤4.0 Å 74.6% of the 40,000 frames, while in rnNOS, this value decreased to 33.1%. The results of the SMD calculations are listed in Figure 6.

While there is variation between each active site in the dimer, approximately 4–6 kcal/mol more force is required to rotate the Tyr “up” in beNOS than in rnNOS. Both computational approaches thus show that the likely reason that some inhibitors can more easily replace the Tyr in rnNOS is due to the greater mobility of this residue in rnNOS.

**Figure 7:**
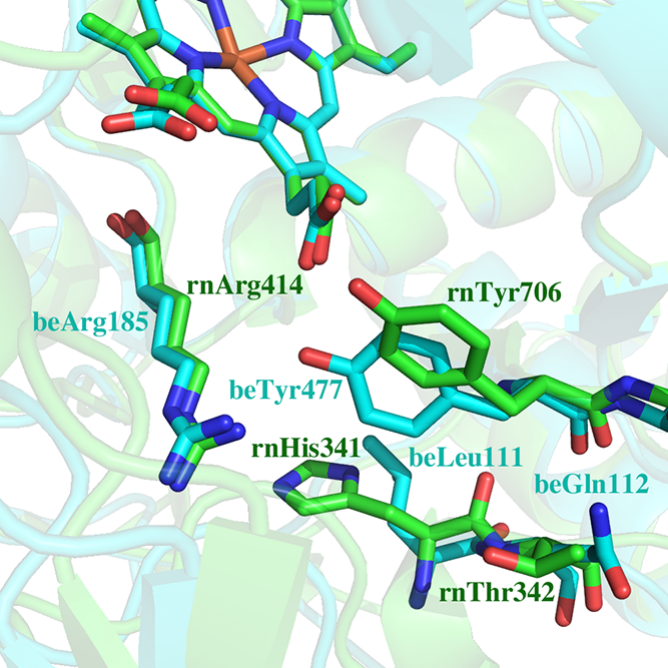
Comparison of rnNOS (green, 6ngl) and beNOS (cyan, 5vv8) in the immediate vicinity of the Tyr that can adopt either the “up” or “down” positions. In rnNOS, His341 prevents Tyr706 from having optimal interactions with Arg414. The residue in beNOS corresponding to rnNOS His341 is Leu111, which enables a better interaction between Tyr477 and Arg185. In addition, Gln112 in beNOS (Thr342 in rnNOS) can form H-bonding interactions with the Tyr477 carbonyl O atom. These differences help stabilize Tyr477 in beNOS at the “down” position. These are the initial energy-minimized models used for MD simulations.

The structural basis for this difference appears to involve several differences between rnNOS and beNOS in the region surrounding the tyrosine, as shown in Figure 7. In rnNOS, His341 is positioned just below Tyr706 while in beNOS, this residue is a leucine (Leu111). Leu111 tucks farther away from Tyr477 enabling the Tyr to be positioned more snuggly between the heme propionate and Arg185 for stronger H-bonding interactions. In contrast, His341 in rnNOS prevents Tyr706 from being similarly positioned. Another possibly important difference is that Thr342 in rnNOS corresponds to Gln112 in beNOS. Gln112 has the potential for H-bonding with the peptide carbonyl oxygen of Tyr706, which could help hold Tyr706 in position. There is no similar possible interaction with Thr342 in rnNOS.

### Dynamics of the Protein Backbone

**Figure 8:**
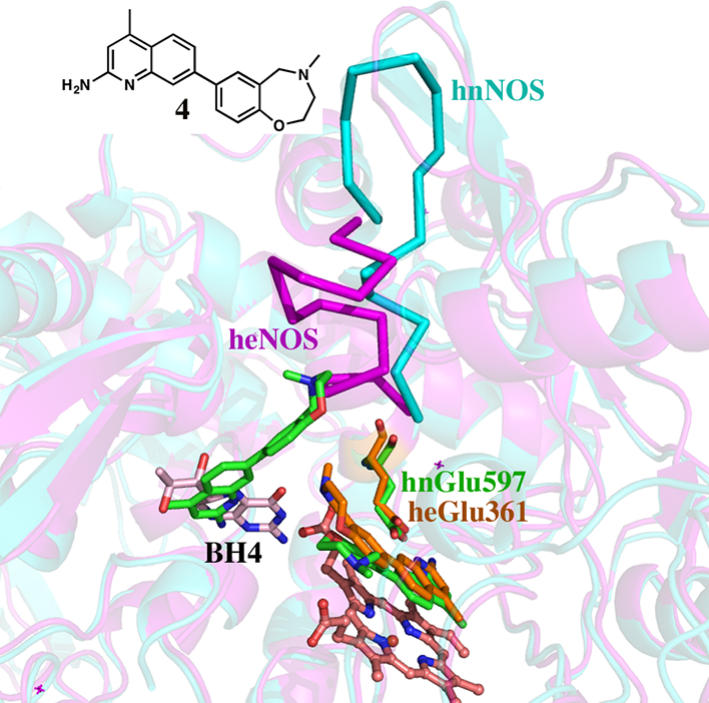
Crystal structure of **4** bound to hnNOS (cyan) and heNOS (magenta). Similar to **3** in hnNOS, there are two molecules of **4** bound in both the active site and the BH_4_ site (green sticks). To accommodate the second **4** molecule, substantial backbone changes in hnNOS occurred, with the region highlighted by the cyan ribbons. In heNOS, only one **4** molecule (orange sticks) binds in the active site and BH_4_ remains in the pterin site. The region in heNOS corresponding to the backbone changes that occurred in hnNOS is highlighted by the magenta ribbon. The same backbone conformation represented by this magenta ribbon would also exist in hnNOS if no inhibitor is bound.

We first observed protein backbone structural changes with compound **3**, followed by observations of the same structural changes in nNOS with many other inhibitors (12 cases in hnNOS and four cases in rnNOS, unpublished results). Compound **4** is a typical example, as shown in Figure 8, where the inhibitor can bind both to the active site and to the BH_4_ site in hnNOS, triggering backbone changes, but only one inhibitor binds to the active site in heNOS and does not induce backbone changes. We next carried out more directed studies to try to elucidate this difference in isoform dynamics.

**Figure 9:**
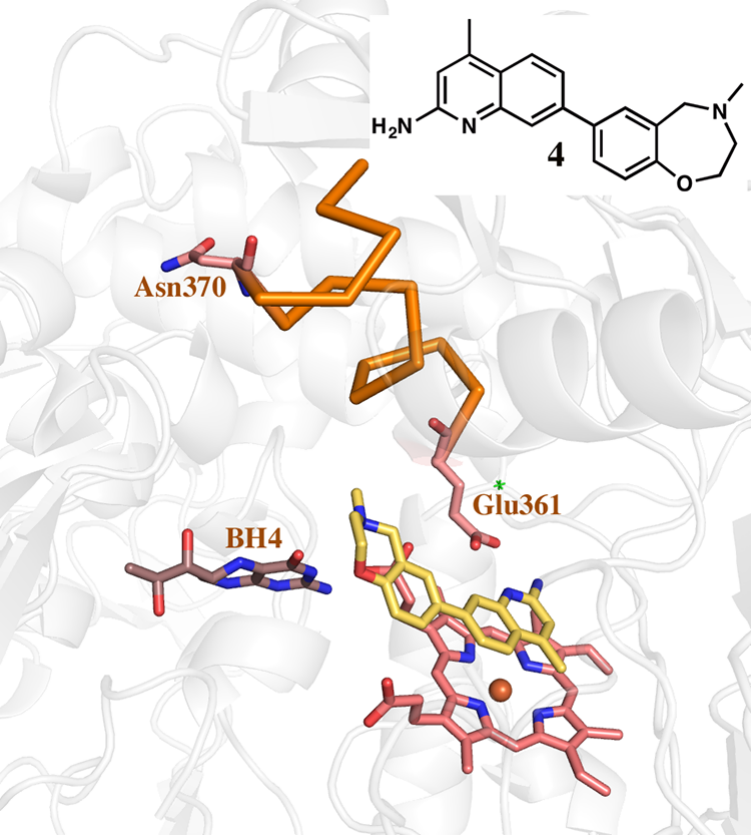
Crystal structure of the heNOS P370N mutant with **4** bound. One molecule of **4** binds in the active site and BH_4_ is undisturbed. The backbone region highlighted by the orange ribbon is unchanged as compared with the wild-type and the unbound heNOS structures.

First, we examined the sequence variations between hnNOS and heNOS in the region Ile598–Ile611, where the backbone changes occur in hnNOS. The position of Asn606 in hnNOS corresponds to Pro370 in heNOS (Figure 3B). The rigidity of the Pro residue could be one reason that heNOS resists undergoing backbone changes upon inhibitor binding. Two reverse mutations were made, hnNOS N606P and heNOS P370N, to test this idea. Unfortunately, we were unable to obtain diffraction-quality crystals with the hnNOS N606P mutant. However, the crystal structure of heNOS P370N bound with **4** (Figure 9) revealed that the inhibitor still binds only at the active site while BH_4_ is undisturbed, just as in the wild-type heNOS-**4** complex (Figure 8).

Since the single residue variation between Asn606 in hnNOS and Pro370 in heNOS does not appear to be the reason for the backbone dynamic differences, we next analyzed the connection between BH_4_ binding and the structural changes induced by inhibitor binding in this region. An Arg residue, Arg601 in hnNOS or Arg365 in heNOS, normally provides a water-mediated H-bond to BH_4_. When BH_4_ is displaced by an inhibitor, this Arg residue is free to undergo the structural change observed in hnNOS. We asked the questions, how can the inhibitor displace BH_4_, and does the inhibitor displacing BH_4_ in hnNOS bind synergistically or independently from the inhibitor bound in the active site?

**Figure 10:**
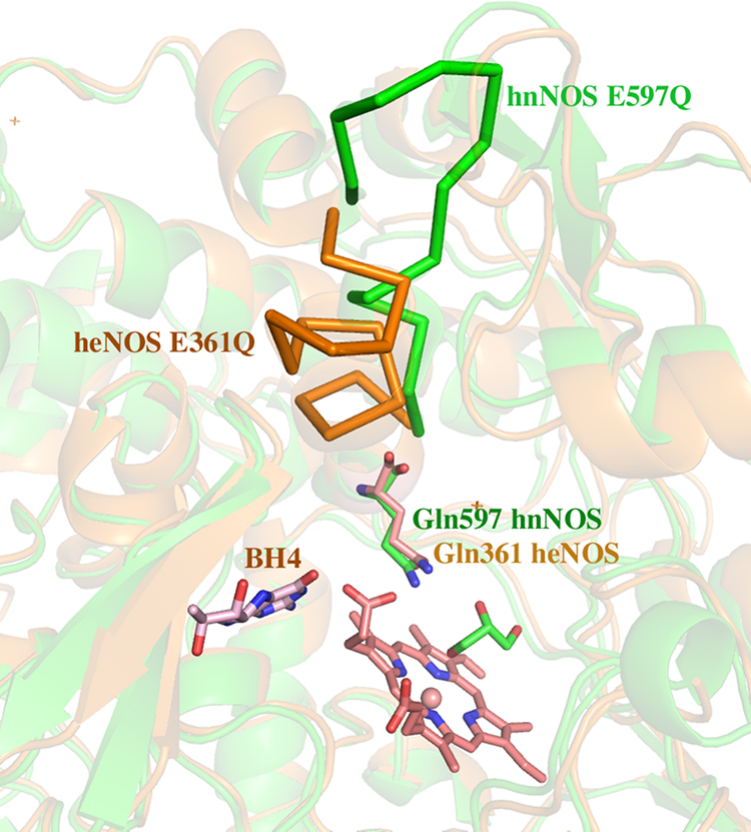
Crystal structures of hnNOS E597Q (green) and heNOS E361Q (orange) obtained with crystals soaked with compound **4**. No inhibitor was bound to the active site in either structure. Electron density in the active site of hnNOS E597Q was modeled as glycerol. The region of residues 598–611 in the hnNOS E597Q mutant undergoes backbone changes highlighted with green ribbon that are the same as those observed in the wild-type hnNOS-**4** structure (Figure 8). There is no backbone change in heNOS E361Q in the region highlighted with an orange ribbon.

To explore these questions, we “knocked out” the active site inhibitor binding by mutating the active site Glu to Gln in both hnNOS and heNOS to generate hnNOS E597Q and heNOS E361Q. As shown in Figure 10, **4** binds in neither the active site nor the BH_4_ site in these hnNOS and heNOS mutants. This result indicates that the binding of two inhibitor molecules is synergistic and that one molecule must first bind in the active site before a second molecule binds in the BH_4_ pocket. Indeed, in the various structures with two inhibitor molecules bound, the inhibitor bound in the active site contacts the inhibitor in the BH_4_ pocket.^13^

Most unexpected, however, was that the same backbone changes in the region of hnNOS residues 598–611 were observed when two molecules bind to wild-type hnNOS occurred in the compound **4**-soaked hnNOS E597Q structure, even though no inhibitor was bound in the active site and BH_4_ remained bound in its site. Therefore, the simple elimination of one charge in the active site results in this structural change without the need for inhibitor binding. This result underscores the complex interplay between the active site and the region that undergoes the conformational change several Ångströms away.

**Figure 11:**
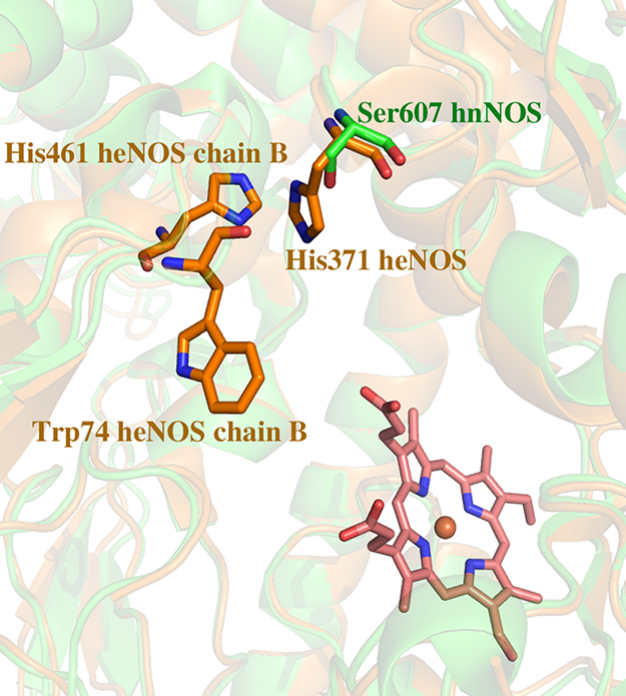
Superposition of the hnNOS (4d1n, green) and heNOS (4dio, orange) crystal structures. Ser607 of hnNOS and its equivalent residue His371 of heNOS are at the dimer interface. In the heNOS structure, His371 can make van der Waals contacts with Trp74 and His461 from the other heNOS subunit. In hnNOS, however, Ser607 is too small to have significant contact with the residues in the neighboring subunit.

It has been shown that eNOS has a stronger dimer interface than nNOS,^32^ and residues 598–611 in hnNOS that undergo the observed conformational change are at the dimer interface. One residue, Ser607 in hnNOS, caught our attention since the equivalent residue in heNOS is a His (His371, Figure 3B). The bulkier His371 in heNOS makes extensive contact with Trp74 and His461 in the neighboring subunit, while Ser607 in hnNOS has no contact at the dimer interface (Figure 11). We therefore hypothesized that the weaker dimer interface interactions in hnNOS allow the 598–611 region a wider range of dynamic flexibility than the corresponding region in heNOS. Our attempts to generate mutants at the interface to test this hypothesis failed since the mutants were too unstable to express and purify.

**Figure 12:**
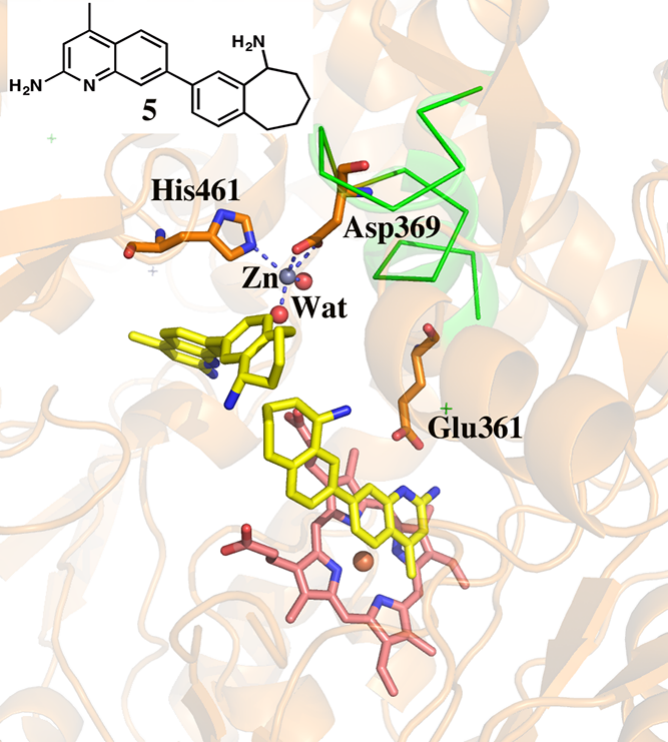
Crystal structure of heNOS with inhibitor **5** bound in both the active site and the BH_4_ site. Displacing BH_4_ results in a new Zn binding site. The helical region highlighted with a green ribbon corresponds to the region that undergoes significant backbone changes in hnNOS. Note that one of the Zn ligands is Asp369 which is in the green region, thus preventing it from moving in heNOS.

It is interesting to note that in some cases, BH_4_ in heNOS can be displaced by an inhibitor. However, instead of undergoing backbone changes in the region 362–375 which corresponds to hnNOS residues 598–611 (Figure 3B), a new Zn site is created where Asp369 and His461 from the other dimer subunit are part of the coordinating ligands, similar to the Zn site observed in the rnNOS-**3** structure (Figure 3A). An example of this is illustrated in the structure of heNOS-**5** (Figure 12). Possibly, the interaction between Asp369 and the Zn atom keeps this region anchored down so that it cannot make a backbone change analogous to that seen in nNOS upon inhibitor binding in the BH_4_ site.

## Conclusions

In this study, we have analyzed differences in the dynamics between nNOS and eNOS upon the binding of various NOS inhibitors. With some inhibitors, we have found that the invariant tyrosine hydrogen bonded to one of the heme propionates is able to move and allows the propionate to bind to the tail end of inhibitors only in nNOS. Our computational results indicate that this tyrosine is anchored in place more tightly in eNOS so that movement of the tyrosine is more energetically costly in eNOS than in nNOS. This is one important difference that can be exploited in developing inhibitors selective for nNOS.

A second major difference is that with some NOS inhibitors, two inhibitor molecules bind to each dimer subunit. One inhibitor molecule is in the active site, as expected, but the second can displace the BH_4_ cofactor in nNOS but is much less likely to do so in eNOS. Inhibitor binding in the BH_4_ site often requires a significant conformational rearrangement near the dimer interface to provide room for the second inhibitor molecules. We initially reasoned that this may be due to the small primary sequence differences between the two NOS isoforms. In the region that changes conformations, there is an Asn in nNOS (Asn606) but a Pro (Pro370) in eNOS. Given the conformational restrictions of Pro, the required conformational change may be more difficult in eNOS. Our mutagenesis results showed that this is not the case.

It appears more likely that the tighter dimer interface in eNOS makes it more difficult to displace BH_4_ since BH_4_ sits at the dimer interface and is likely important for stabilizing the dimer. There is abundant evidence indicating that the eNOS dimer is tighter and more stable than the other isoforms.^32^ It also appears that the eNOS dimer is less dependent on BH_4_ being bound since we have been able to purify and crystallize BH_4_-free eNOS but not BH_4_-free nNOS (results not shown).

In this study, we also found that the binding of a second inhibitor molecule in the BH_4_ pocket first requires the binding of the inhibitor to the active site. In addition, mutating the active site Glu to Gln in nNOS resulted in the same conformational change that results from two inhibitors binding. This suggests that when two inhibitors bind, the first one enters the active site and hydrogen bonds with the active site Glu, lowering the energetic barrier to the conformational change required to bind the second molecule that displaces BH_4_. This surprising result, that changing an active site residue promotes the conformational change at the dimer interface some distance away, underscores the complex interplay between the active site and the dimer interface. These new insights into the dynamical differences between NOS isoforms should prove useful in the development of isoform-selective inhibitors.

## Supporting Information Available

Crystallographic data collection; refinement statistics; and synthetic chemistry (PDF)

## Accession Codes

The coordinates have been deposited in the Protein Data Bank as entries 8UFP, 8UFQ, 8UFR, 8UFS, 8UFT, and 8UFU. The PIR protein database IDs are P29473 beNOS; P29474 heNOS; P29475 hnNOS; P29476 rnNOS.
