# Supplementary material for: Crystallographic and Computational Insights into Isoform-Selective Dynamics in Nitric Oxide Synthase
Source: Biochemistry. 2024 Feb 28;63(6):788–96. doi: 10.1021/acs.biochem.3c00601 (PMC10956423; doi:10.1021/acs.biochem.3c00601)
Supplement: Supplementary file 1 — bi3c00601_si_001.pdf [file bi3c00601_si_001.pdf]

Supporting Information

Crystallographic and Computational Insights into Isoform-Selective Dynamics in Nitric Oxide Synthase

Huiying Li[§], Christine D. Hardy[§], Cory T. Reidl[†,#], Qing Jing[†,£], Fengtian Xue[†,€], Maris Cinelli, [†,‡], Richard B. Silverman[†,¶,*], and Thomas L. Poulos[§,*]

[§] Departments of Molecular Biology and Biochemistry, Pharmaceutical Sciences, and Chemistry, University of California, Irvine, California 92697-3900, United States

[†] Department of Chemistry, Department of Molecular Biosciences, Chemistry of Life Processes Institute, Center for Developmental Therapeutics, Northwestern University, 2145 Sheridan Road, Evanston, Illinois 60208-3113, United States

[¶] Department of Pharmacology, Feinberg School of Medicine, Northwestern University, Chicago, Illinois 60611, United States

[#] Current address: Evonik Corporation, Tippecanoe Laboratories 1650 Lilly Rd., Lafayette, IN 47909, United States

[£] Current address: Novartis Pharmaceutical Corporation, 6201 South Fwy, CC/BC 2, Fort Worth, TX 76134-2009, United States

[€] Current address: Department of Pharmaceutical Sciences, University of Maryland, Baltimore, MD 21201

[‡] Current Address: Department of Chemistry, Northern Michigan University, 1401 Presque Isle, Marquette, MI 49855-5301

Corresponding Authors

*Thomas L. Poulos -- poulos@uci.edu

*Richard B. Silverman -- Agman@chem.northwestern.edu

Table S1. Crystallographic data collection and refinement statistics

Data set[a]	hnNOS-**4**	hnNOS E597Q-**4**	heNOS-**4**
**Data collection**			
PDB code	8UFP	8UFQ	8UFR
Space group	$P2_12_12_1$	$P2_1$	$P2_1$
Cell dimensions			
a, *b*, *c* (Å)	52.1 122.1 164.9	117.0 51.6 162.3	59.5 152.9 108.9
β (º)	-	90.00	90.86
Resolution (Å)	1.90 (1.90-1.94)	1.98 (1.98-2.01)	1.87 (1.87-1.90)
R_{merge}	0.184 (2.972)	0.147 (2.192)	0.122 (4.185)
R_{pim}	0.059 (0.939)	0.076 (1.130)	0.062 (2.247)
$R_{measure}$	0.193 (3.120)	0.166 (2.474)	0.137 (4.775)
CC 1/2	0.997 (0.769)	0.995 (0.407)	0.995 (0.285)
$< I / \sigma I >$	7.6 (1.42)[c]	6.6 (1.09)[c]	6.3 (1.50)[c]
No. unique reflections	83613 (4568)	133216 (6450)	157245 (7360)
Completeness (%)	99.9 (99.7)	99.5 (98.1)	98.0 (93.1)
Redundancy	10.5 (10.9)	4.6 (4.6)	4.7 (4.2)
Wilson B factor (Å)	27.7	31.4	34.5
**Refinement**			
Resolution (Å)	1.90	1.98	1.87
No. reflections used	83216	133189	146488
R_{work} / R_{free}^{b}	0.195/0.237	0.181/0.228	0.207/0.251
No. atoms, Protein	6786	13669	12820
Ligand/ion	211	202	542
Water	703	1193	468
R.m.s. deviations			
Bond lengths (Å)	0.007	0.007	0.010
Bond angles (º)	0.94	0.95	1.09

Data set[a]	heNOS E361Q-**4**	heNOS P350N-**4**	heNOS-**5**
**Data collection**			
PDB code	8UFS	8UFT	8UFU
Space group	$P2_1$	$P2_1$	$P2_1$
Cell dimensions			
a, *b*, *c* (Å)	59.8 152.2 108.5	60.1 154.6 108.6	59.7 152.8 109.2
β (º)	90.78	90.73	90.82
Resolution (Å)	2.05 (2.05-2.09)	1.78 (1.81-1.78)	2.05 (2.08-2.05)
R_{merge}	0.144 (1.194)	0.119 (2.086)	0.159 (4.131)
R_{pim}	0.096 (0879)	0.060 (1.088)	0.084 (2.242)
$R_{measure}$	0.174 (1.493)	0.133 (2.361)	0.180 (3.716)
CC 1/2	0.985 (395)	0.996 (0.488)	0.992 (0.312)
$< I / \sigma I >$	4.8 (1.58)	9.0 (1.80) [c]	5.4 (1.40) [c]
No.unique reflections	118594 (5663)	186583 (9137)	118090 (5346)
Completeness (%)	97.8 (94.3)	98.8 (97.9)	96.5 (88.8)
Redundancy	3.1 (2.8)	4.7 (4.3)	4.4 (4.2)
Wilson B factor (Å)	27.9	25.6	35.92

**Refinement**			
Resolution (Å)	2.05	1.78	2.05
No. reflections used	115538	185007	115990
R_{work} / R_{free}^{b}	0.185/0.235	0.175/0.210	0.205/0.256
No. atoms, Protein	12826	12931	12820
Ligand/ion	460	523	539
Water	716	1319	362
R.m.s. deviations			
Bond lengths (Å)	0.009	0.012	0.014
Bond angles (º)	0.98	0.95	1.02

[a] See Schemes 1 and 2 for nomenclature and chemical formula of inhibitors.

[b] R_{free} was calculated with the 5% of reflections set aside throughout the refinement. The set of reflections for the R_{free} calculation were kept the same for all data sets according to those used in the data of the starting model (4UH5 for hnNOS in $P2_12_12_1$, 7TS7 for hnNOS in $P2_1$, and 5UO8 for heNOS).

[c] The $< I / \sigma I >$ value in the highest resolution shell calculated by Xtriage and reported in the wwPDB X-ray Structure Validation Report.

Synthetic Chemistry

7-chloro-4-methyl-2,3,4,5-tetrahydrobenzo[f][1,4]oxazepine (CTR-1-172-B). To a 50 mL, 2-neck round bottom flask equipped with a magnetic stir bar was charged chlorobenzooxazepine (0.402 g, 1.83 mmol) and paraformaldehyde (0.55 g, 18 mmol). The flask was then charged with AcOH (9.2 mL) and degassed with nitrogen gas for 5 minutes while stirring over an ice bath. $NaBH_3CN$ (0.575 g, 9.15 mmol) was then added in multiple portions to the reaction mixture to avoid an exothermic runaway reaction. The vessel was sealed with a septum and allowed to stir for 5 hours at room temperature, with the reaction progress being monitored periodically by TLC. After completion of the reaction, the mixture was quenched by adding solid excess 2M NaOH. The product was extracted 3x with 20 mL of CH_2Cl_2. The organic layers were combined and concentrated to dryness using a rotary evaporator. The residue was diluted with 10 mL of pure hexane and stirred for 5-10 minutes, followed by filtering through celite. The filtrate was concentrated onto celite, loaded into a dry load cartridge, and purified by normal phase flash chromatography (12 g RediSep Gold® Silica Gel Disposable Flash Columns), eluting with a gradient of CH_2Cl_2 to EA to afford 0.32 g of the desired product as a white solid in 75% yield. ^{1}H NMR (500 MHz, CD_3OD) δ 7.50 (d, J = 2.6 Hz, 1H), 7.43 (dd, J = 8.6, 2.6 Hz, 1H), 7.15 (d, J = 8.6 Hz, 1H), 4.57 (d, J = 13.8 Hz, 1H), 4.50 (d, J = 12.0 Hz, 2H), 4.17 – 4.08 (m, 1H), 3.83 – 3.76 (m, 1H), 3.74 – 3.65 (m, 1H), 3.06 (s, 3H). ^{13}C NMR (126 MHz, CD_3OD) δ 159.0, 131.5, 131.1, 129.1, 124.8, 122.6, 67.7, 58.8, 57.6, 42.2.

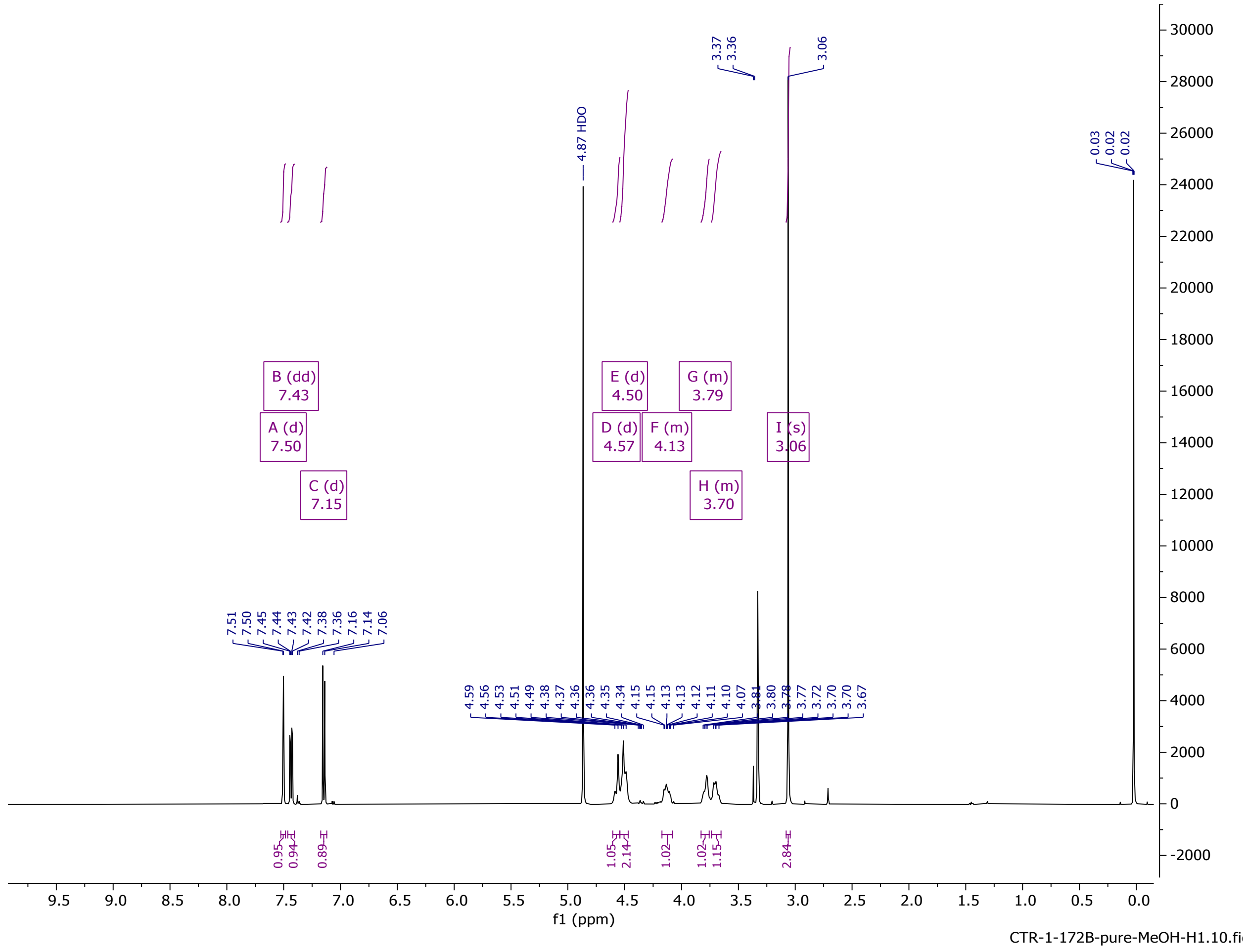

Figure S1. ^{1}H NMR for **7-chloro-4-methyl-2,3,4,5-tetrahydrobenzo[f][1,4]oxazepine (CTR-1-172-B)**.

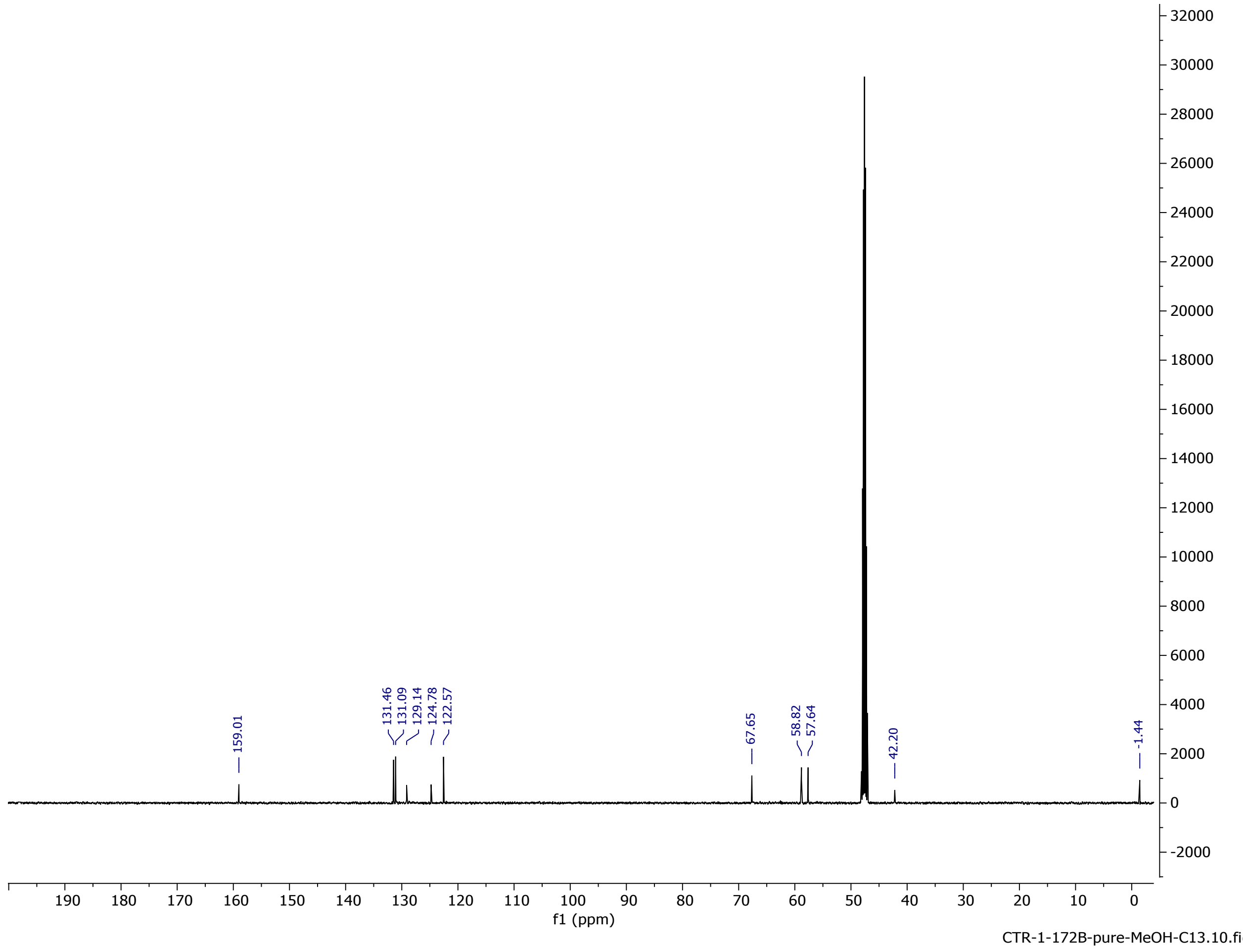

Figure S2. ^{13}C NMR for **7-chloro-4-methyl-2,3,4,5-tetrahydrobenzo[f][1,4]oxazepine (CTR-1-172-B)**.

N-(4-methyl-7-(4-methyl-2,3,4,5-tetrahydrobenzo[f][1,4]oxazepin-7-yl)quinolin-2-yl)acetamide (CTR-1-175). In a 20 mL glass vial equipped with stir bar and septum cap was added XPhos-Pd-G3 (2.0 mg, 2.4 μmol), XPhos (2.3 mg, 4.8 μmol), tetrahydroxydiboron (65.0 mg, 0.73 mmol), and KOAc (95.0 mg, 0.97 mmol). EtOH (0.1 vol.) was charged followed by the addition of the **CTR-1-172-B** (57.0 mg, 0.24 mmol). The reaction mixture was sealed under argon and heated to 80 °C for 2 hours. The reaction was then charged with 3 equivalents of degassed 1.8 M aqueous K_2CO_3 (0.4 mL,

0.73 mmol) followed by **N-(7-bromo-4-methylquinolin-2-yl)acetamide** (65.6 mg, 0.24 mmol).[1-2] The reaction mixture was flushed with argon and heated to 80 °C overnight. The reaction mixture was cooled to room temperature, diluted with water (5-10 mL) and extracted with EA (3 × 10 mL). The combined organics were dried over anhydrous Na_2SO_4 and concentrated under a nitrogen stream. The crude product compound was purified by normal phase flash column chromatography (12 g RediSep Gold® Silica Gel Disposable Flash Columns), eluting with a gradient of 100% CH_2Cl_2 to 100% EA, then to 2% MeOH-EA, to afford 61.9 mg of **CTR-1-175** as crystalline colorless solid in 72% yield. This material was used without further purification. ^{1}H NMR (500 MHz, DMSO) δ 10.69 (s, 1H), 8.15 (s, 1H), 8.06 (d, *J* = 8.6 Hz, 1H), 7.94 (d, *J* = 1.9 Hz, 1H), 7.79 (dd, *J* = 8.6, 1.9 Hz, 1H), 7.68 (d, *J* = 2.5 Hz, 1H), 7.62 (dd, *J* = 8.2, 2.4 Hz, 1H), 7.09 (d, *J* = 8.2 Hz, 1H), 4.07 – 4.02 (m, 1H), 3.78 (s, 2H), 3.17 (s, -1H), 2.94 – 2.89 (m, 2H), 2.68 (s, 3H), 2.34 (s, 3H), 2.16 (s, 3H), 1.84 (s, 1H). ^{13}C NMR (126 MHz, DMSO) δ 170.3, 160.3, 152.4, 147.2, 146.5, 141.3, 134.7, 132.8, 129.9, 127.4, 125.2, 125.0, 124.5, 124.1, 121.6, 114.7, 70.8, 61.0, 60.4, 49.1, 44.2, 24.6, 19.2.

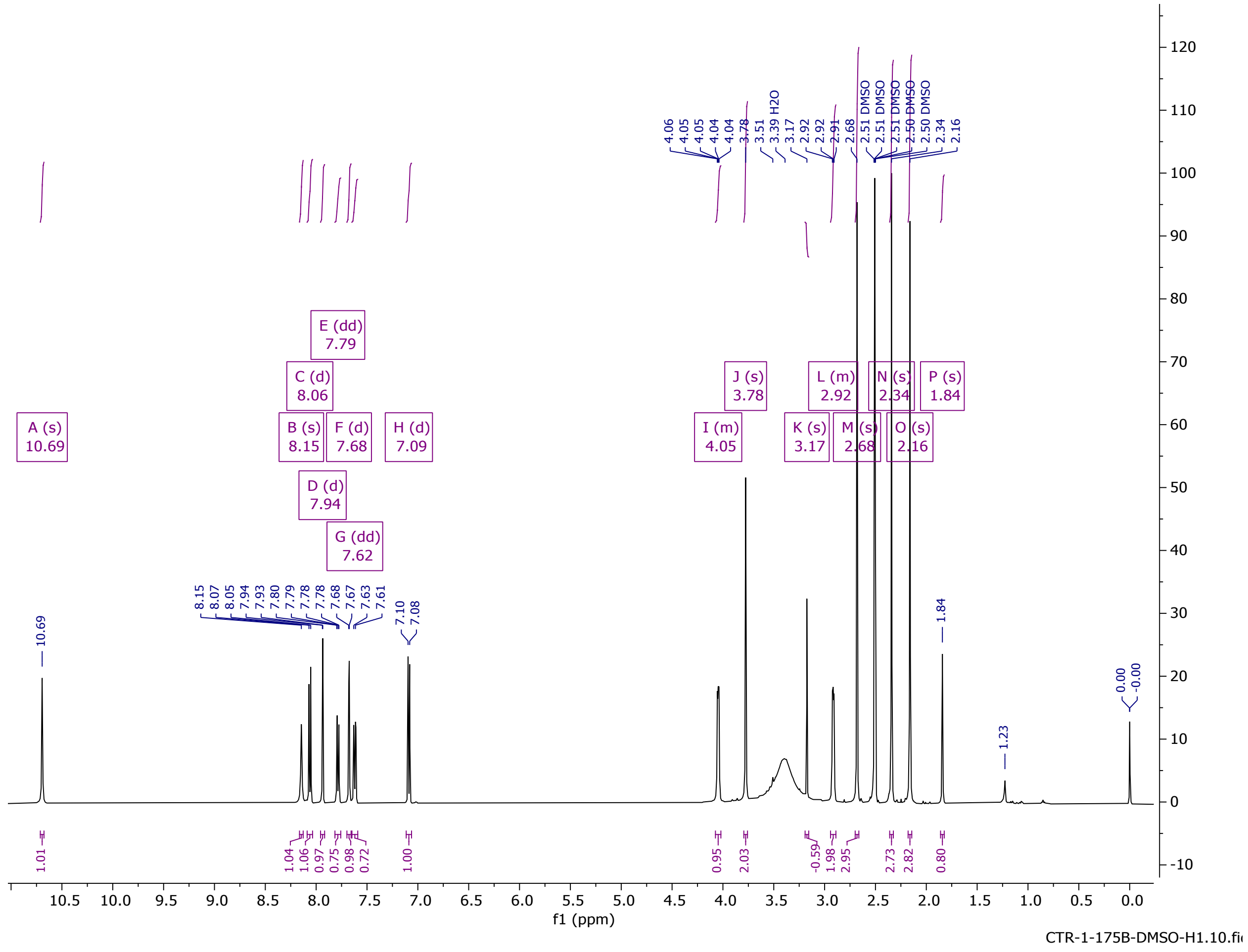

Figure S3. ^{1}H NMR for **N-(4-methyl-7-(4-methyl-2,3,4,5-tetrahydrobenzo[f][1,4]oxazepin-7-yl)quinolin-2-yl)acetamide (CTR-1-175).**

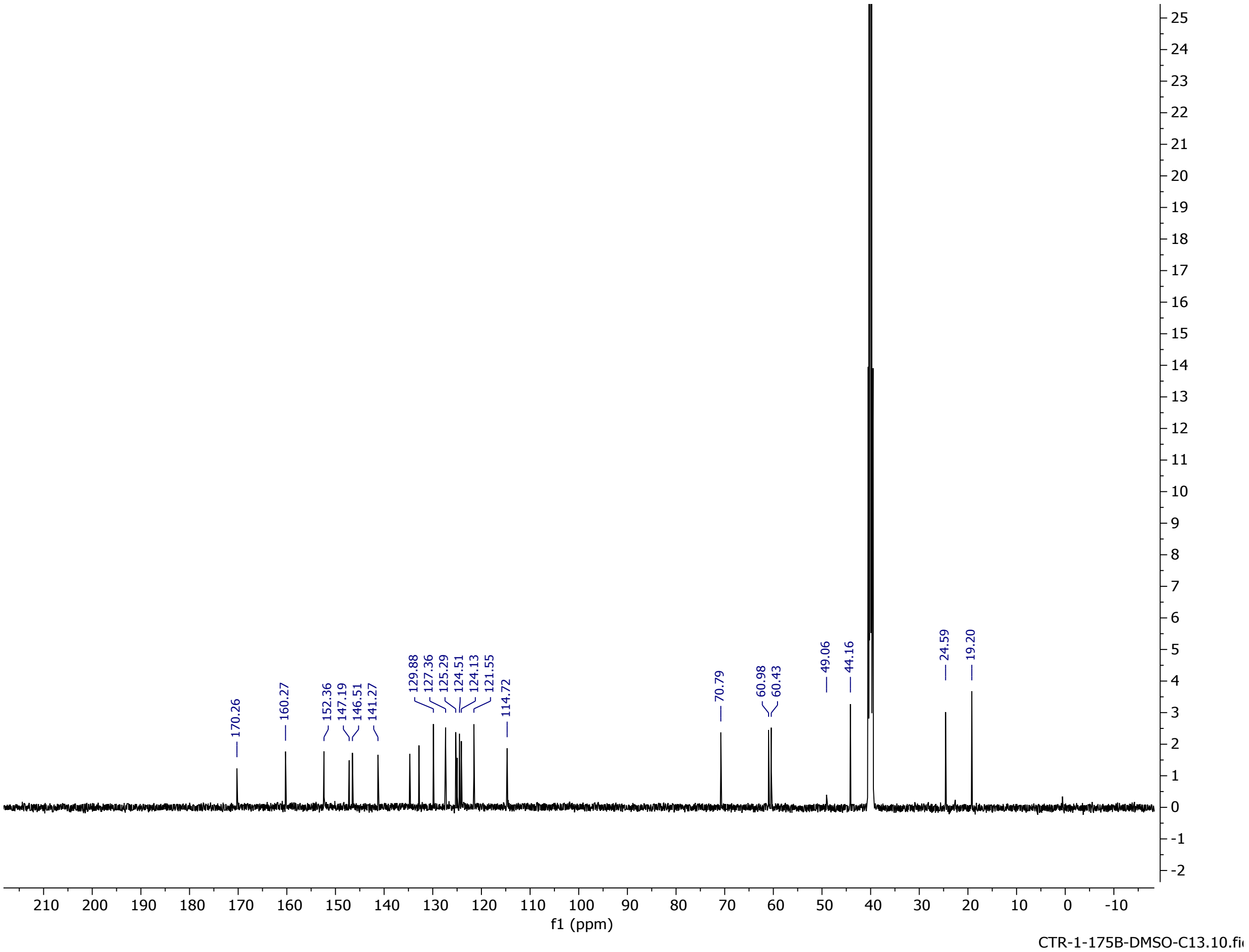

Figure S4. ^{13}C NMR for **N-(4-methyl-7-(4-methyl-2,3,4,5-tetrahydrobenzo[f][1,4]oxazepin-7-yl)quinolin-2-yl)acetamide (CTR-1-175).**

4-Methyl-7-(4-methyl-2,3,4,5-tetrahydrobenzo[f][1,4]oxazepin-7-yl)quinolin-2-amine Dihydrochloride (CTR-3-177-A). The protected intermediate **CTR-1-175** (54.5 mg, 0.15 mmol) was diluted with MeOH (7.6 mL) in a 20 mL vial and charged with K_2CO_3 (41.7 mg, 0.30 mmol) and a stir bar. The mixture was heated at reflux for 2-2.5 h, cooled, and concentrated, and the residue was partitioned between EA (10 mL) and H_2O/sat. aq. NaCl (1:1, 10 mL). The layers were separated and the aqueous phase was extracted with

EA (3 x 10 mL). The combined organics were dried over anhydrous Na_2SO_4, and concentrated. The resulting crude free 2-aminoquinoline residue was treated with 3M methanolic HCl (1 mL) and concentrated to afford **5** in 97% purity by LCMS. The resulting residue was further purified by reverse phase chromatography (15.5 g RediSep Rf Gold® C18 Chromatography Column), eluting with water to afford **5** as a colorless solid in 54% yield after drying under vacuum. MP = 280.4 °C (softens), >294.1 °C decomposed. ^{1}H NMR (500 MHz, DMSO-d_6) δ 14.33 (s, 1H), 11.48 (s, 1H), 8.08 (dd, J = 8.5, 2.0 Hz, 1H), 7.91 – 7.89 (m, 1H), 7.89 (d, J = 2.4 Hz, 1H), 7.78 (dq, J = 8.5, 1.8 Hz, 2H), 7.26 (dd, J = 8.4, 1.5 Hz, 1H), 6.96 (s, 1H), 7.01 – 6.90 (m, 1H), 4.58 (s, 2H), 4.46 (s, 1H), 4.24 (s, 1H), 3.64 (s, 2H), 2.89 (s, 3H), 2.65 (s, 3H). ^{13}C NMR (126 MHz, DMSO) δ 159.6, 153.3, 151.5, 141.9, 135.6, 133.5, 130.5, 128.9, 125.7, 123.7, 122.7, 121.1, 119.7, 113.9, 111.9, 66.7, 56.9, 56.4, 41.0, 18.3. HRMS: calcd. for $C_{20}H_{22}N_3O^+$ 320.1757, found 320.1752.

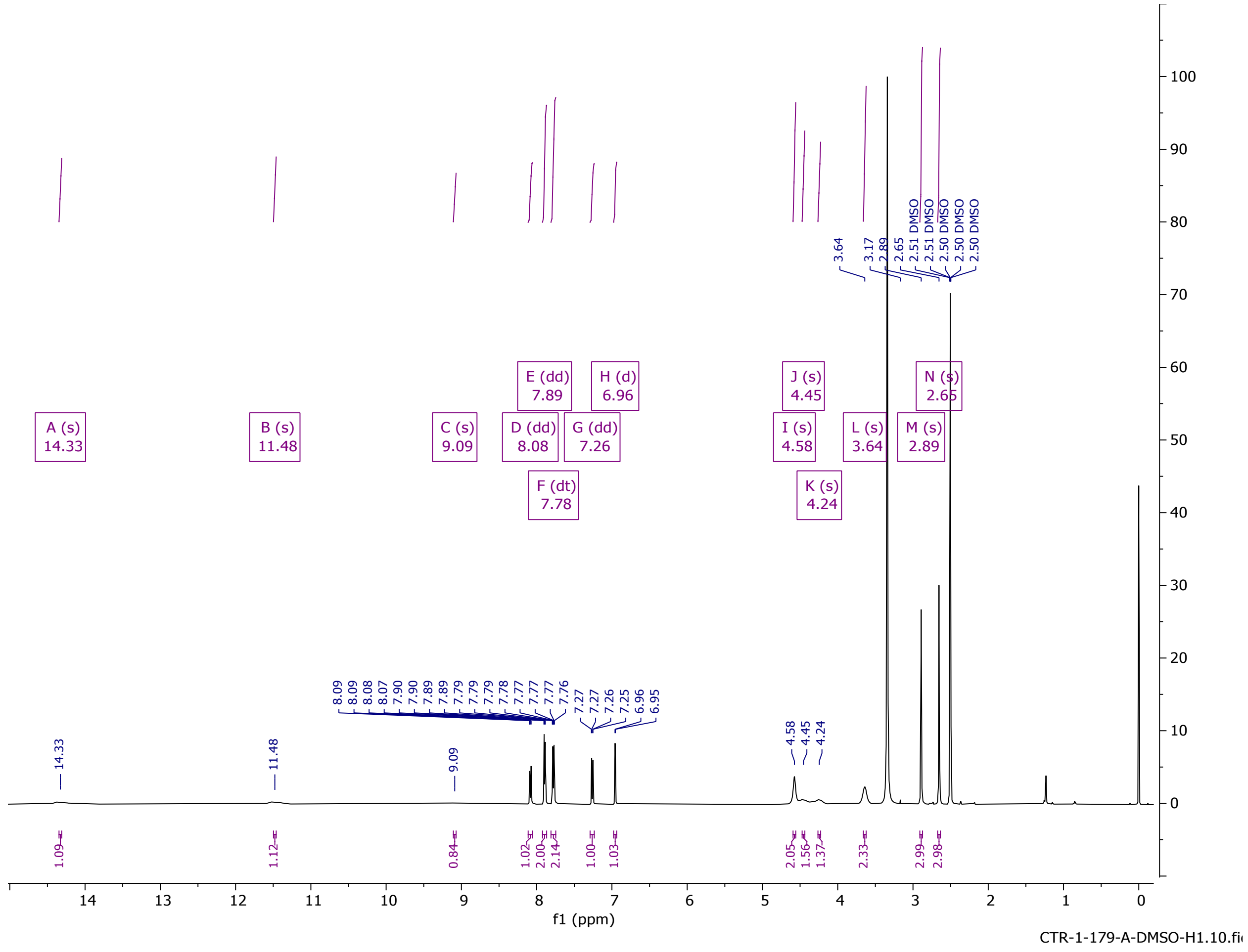

Figure S5. ^{1}H NMR for **4-Methyl-7-(4-methyl-2,3,4,5-tetrahydrobenzo[f][1,4]oxazepin-7-yl)quinolin-2-amine Dihydrochloride (CTR-3-177-A)**.

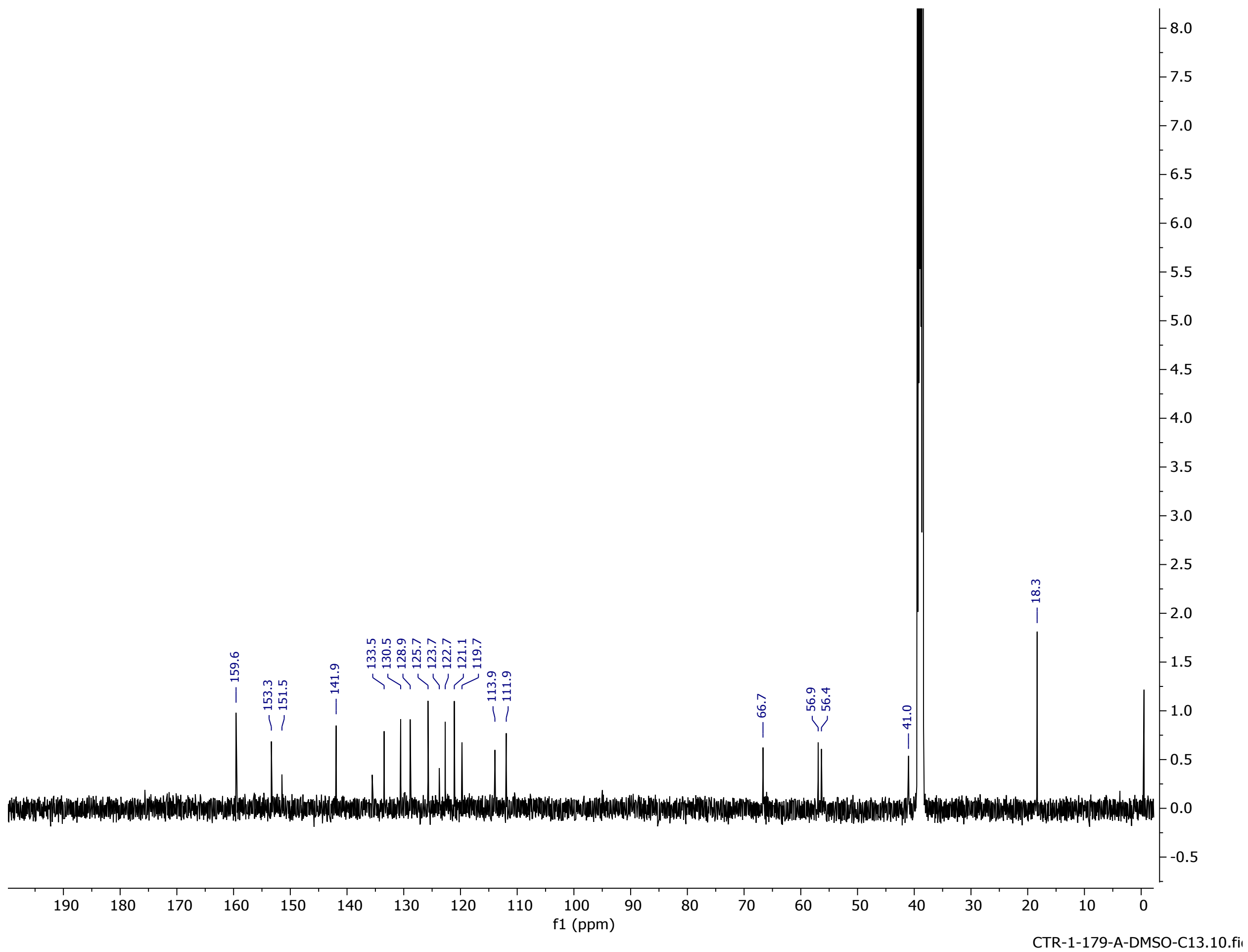

Figure S6. ^{13}C NMR for **4-Methyl-7-(4-methyl-2,3,4,5-tetrahydrobenzo[f][1,4]oxazepin-7-yl)quinolin-2-amine Dihydrochloride (CTR-3-177-A)**.

(*S*)-N-((*S*)-3-bromo-6,7,8,9-tetrahydro-5H-benzo[7]annulen-5-yl)-2-methylpropane-2-sulfinamide and (*S*)-N-((*R*)-3-bromo-6,7,8,9-tetrahydro-5H-benzo[7]annulen-5-yl)-2-methylpropane-2-sulfinamide (CTR-2-172-T17-19 and CTR-2-172-T22-24).

Reaction was performed in a 2-5 mL microwave vial under an argon atmosphere. To the vial was added sealed vial (*S*)-2-methylpropane-2-sulfinamide (0.26 g, 2.2 mmol) and 3-

bromo-6,7,8,9-tetrahydro-5H-benzo[7]annulen-5-one (0.43 g, 1.8 mmol), and a stir bar. The vessel was sealed and flushed with Ar before dry THF (4.5 mL) followed by titanium(V) isopropoxide (1.1 mL, 3.6 mmol) were added via syringe. The reaction shaken to suspend any solids before stirring for 4h at 100 °C under microwave irradiation. The reaction was then held at RT overnight. LCMS analysis the following next morning shows mostly the imine had formed and several minor byproduct peaks. The reaction was cooled in an ice bath for 5 min while freshly crushed $NaBH_4$ tablets (0.34 g, 9.0 mmol) were added to a separate nitrogen flushed 100 mL RBF. The cooled reaction mixture was then added to the RBF reduction vessel via glass pipet, and the microwave vial was subsequently rinsed 2 x with ~2 mL portions of fresh THF. The reaction was allowed to stir under nitrogen at RT for 2h upon which LCMS analysis indicated consumption of imine intermediate and the presence of a new peak corresponding to desired product. The crude mixture was then diluted with 50 mL EA and 50 mL saturated $NaHCO_3$ solution which formed vigorous effervescence as well as white precipitate presumed to be TiO_2. The product slurry was then passed through a celite plug to remove the precipitated TiO_2 and the wet cake was washed several times with fresh EA. The aqueous layer in the biphasic filtrate was then removed and product-rich organic layer was removed and washed with once with 20 mL brine. The organic layer was dried over NaSO4 and concentrated to dryness. The crude residue was purified by normal phase chromatography (12 g RediSep Gold® Silica Gel Disposable Flash Columns), eluting with CH_2Cl_2 to EA gradient, to afford the two diastereomers as closely eluting product peak (Diasteriomer A and B, respectively). The two diastereomers were isolated and kept separate, yielding 0.076 g of Diasteromer A **(CTR-2-172-T17-19)**, the

first peak to elute from the column, as a colorless oil in 12% yield, and 0.112 g of Diasteromer B **(CTR-2-172-T22-24)**, the second peak to elute from the column, as a crystalline solid in 19% yield.

CTR-2-172-T17-19: ^{1}H NMR (500 MHz, $CDCl_3$) δ 7.41 (d, J = 2.2 Hz, 1H), 7.29 (dd, J = 8.0, 2.1 Hz, 1H), 6.97 (d, J = 8.0 Hz, 1H), 4.57 (dt, J = 7.2, 2.3 Hz, 1H), 3.34 (d, J = 2.5 Hz, 1H), 2.99 (ddd, J = 14.4, 10.6, 1.8 Hz, 1H), 2.71 – 2.63 (m, 1H), 2.13 (t, J = 9.1 Hz, 1H), 2.06 – 1.93 (m, 1H), 1.93 – 1.84 (m, 1H), 1.84 – 1.70 (m, 2H), 1.53 (tdd, J = 13.0, 9.3, 5.1 Hz, 1H), 1.27 (s, 9H). ^{13}C NMR (126 MHz, $CDCl_3$) δ 143.9, 141.7, 131.9, 131.0, 130.6, 119.8, 57.9, 56.0, 53.4, 35.0, 31.6, 27.7, 25.8, 22.8.

CTR-2-172-T22-24: ^{1}H NMR (500 MHz, $CDCl_3$) δ 7.48 (d, J = 2.2 Hz, 1H), 7.30 – 7.25 (m, 1H), 6.97 (d, J = 8.0 Hz, 1H), 4.60 (ddd, J = 8.3, 4.0, 1.9 Hz, 1H), 3.29 (d, J = 4.1 Hz, 1H), 2.89 (ddd, J = 14.3, 8.5, 2.5 Hz, 1H), 2.72 (ddd, J = 14.3, 8.9, 2.5 Hz, 1H), 2.00 (qd, J = 7.8, 3.3 Hz, 1H), 1.94 (s, 1H), 1.97 – 1.81 (m, 2H), 1.66 (d, J = 9.0 Hz, 1H), 1.26 (s, 9H). ^{13}C NMR (126 MHz, CDCl3) δ 143.7, 140.9, 131.6, 130.4, 130.1, 119.7, 58.4, 55.8, 35.0, 27.4, 27.3, 22.7.

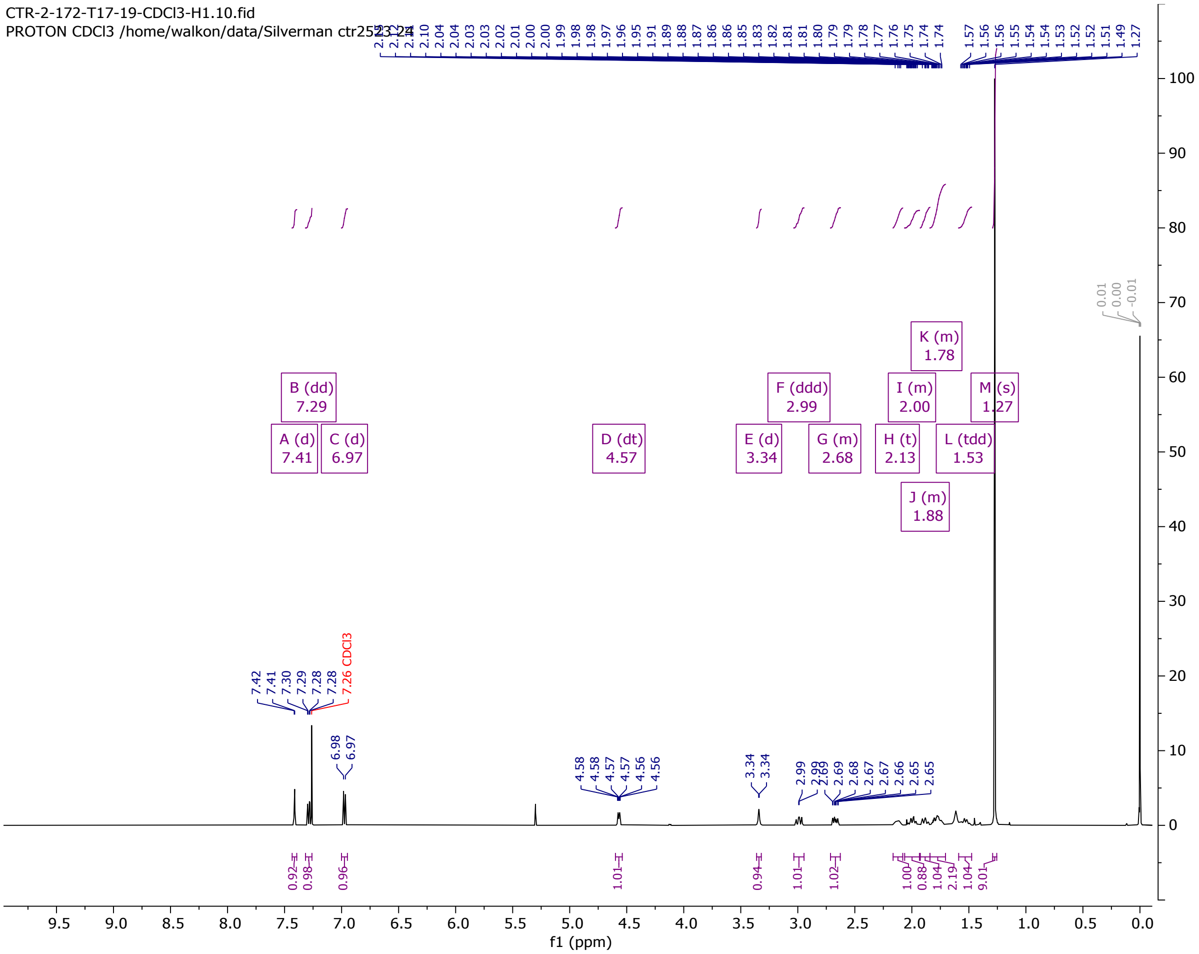

Figure S7. ^{1}H NMR for **(*S*)-N-((*S*)-3-bromo-6,7,8,9-tetrahydro-5H-benzo[7]annulen-5-yl)-2-methylpropane-2-sulfinamide (CTR-2-172-T17-19).**

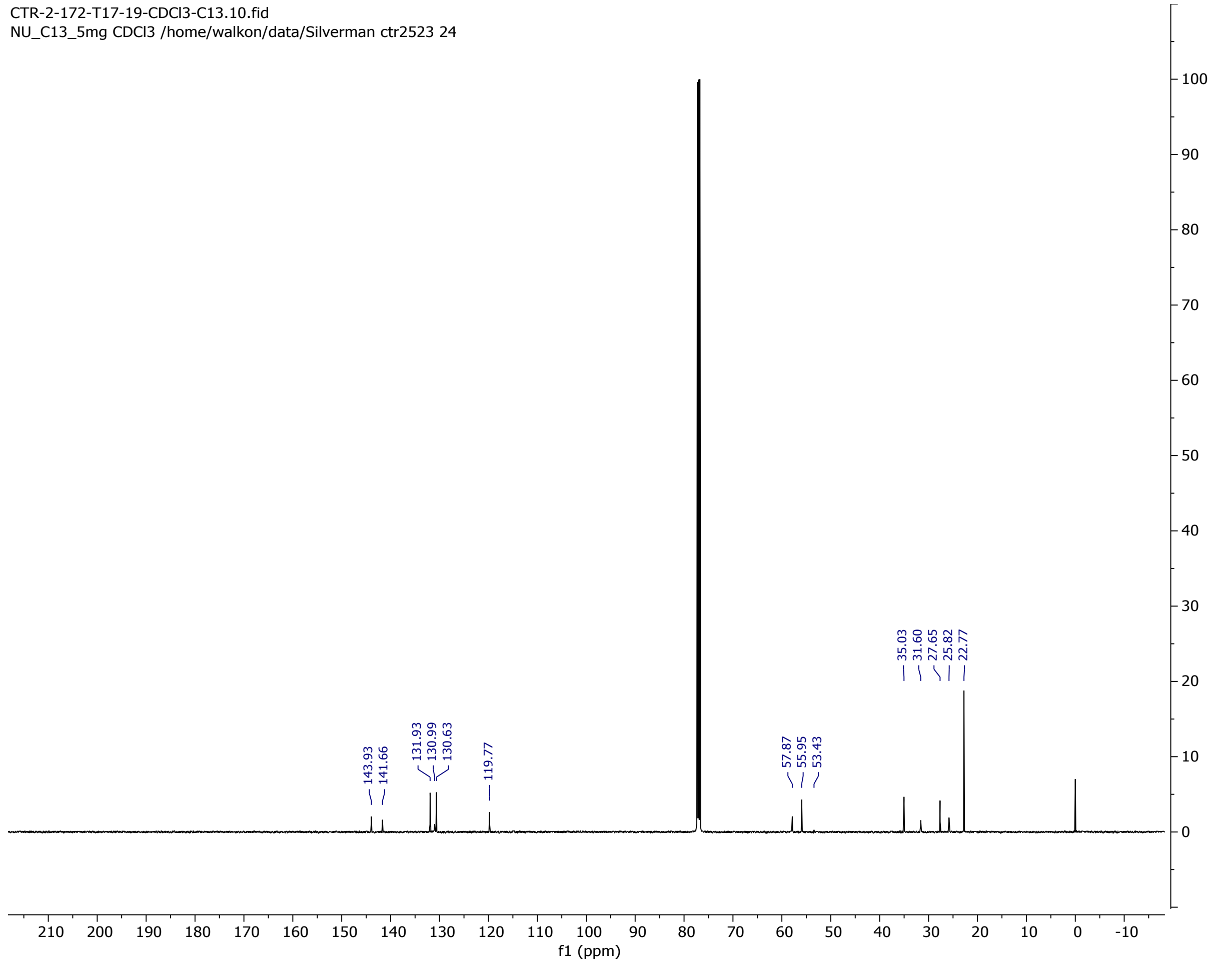

Figure S8. ^{13}C NMR for **(*S*)-N-((*S*)-3-bromo-6,7,8,9-tetrahydro-5H-benzo[7]annulen-5-yl)-2-methylpropane-2-sulfinamide (CTR-2-172-T17-19).**

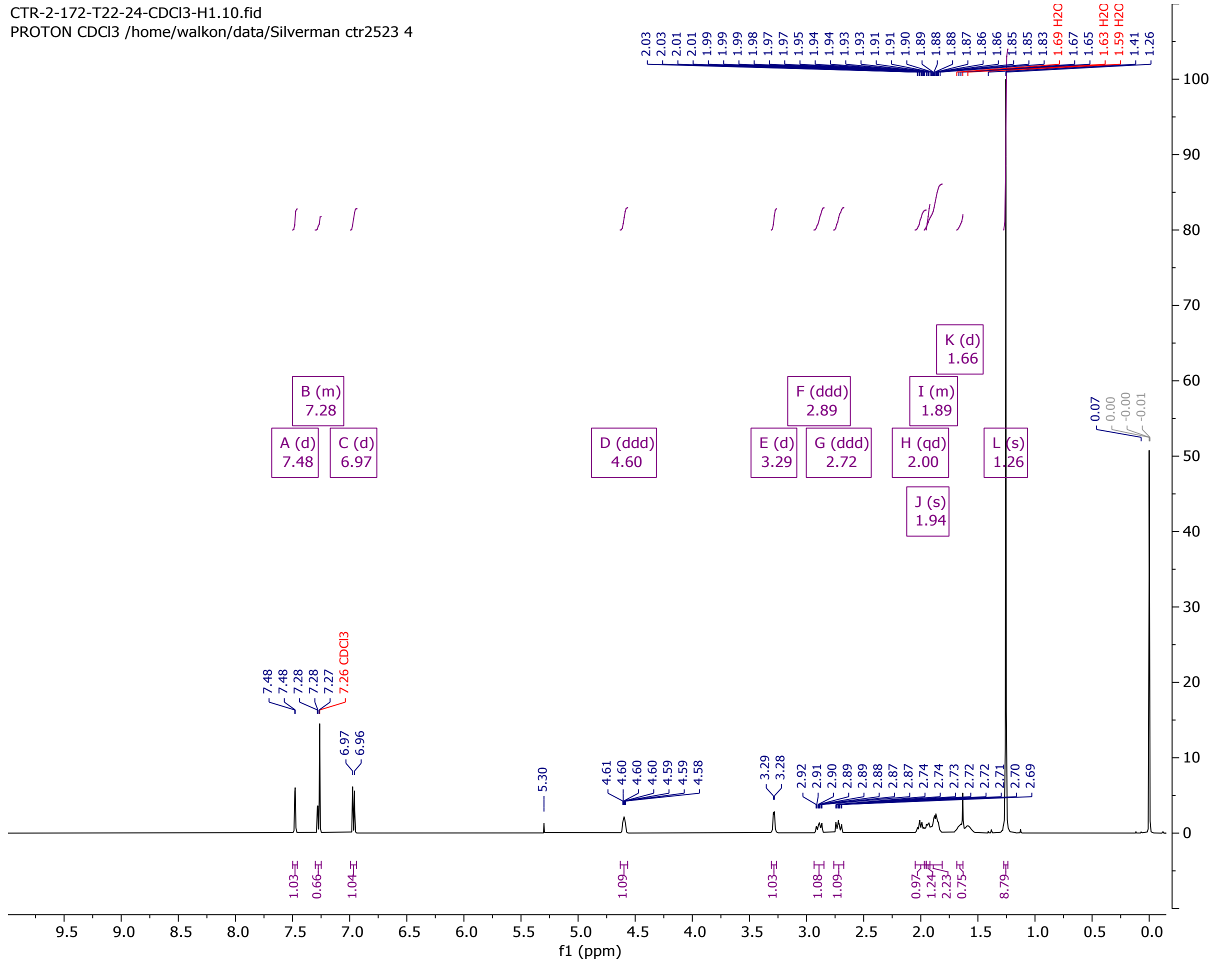

Figure S9. ^{1}H NMR for **(*S*)-N-((*R*)-3-bromo-6,7,8,9-tetrahydro-5H-benzo[7]annulen-5-yl)-2-methylpropane-2-sulfinamide (CTR-2-172-T22-24).**

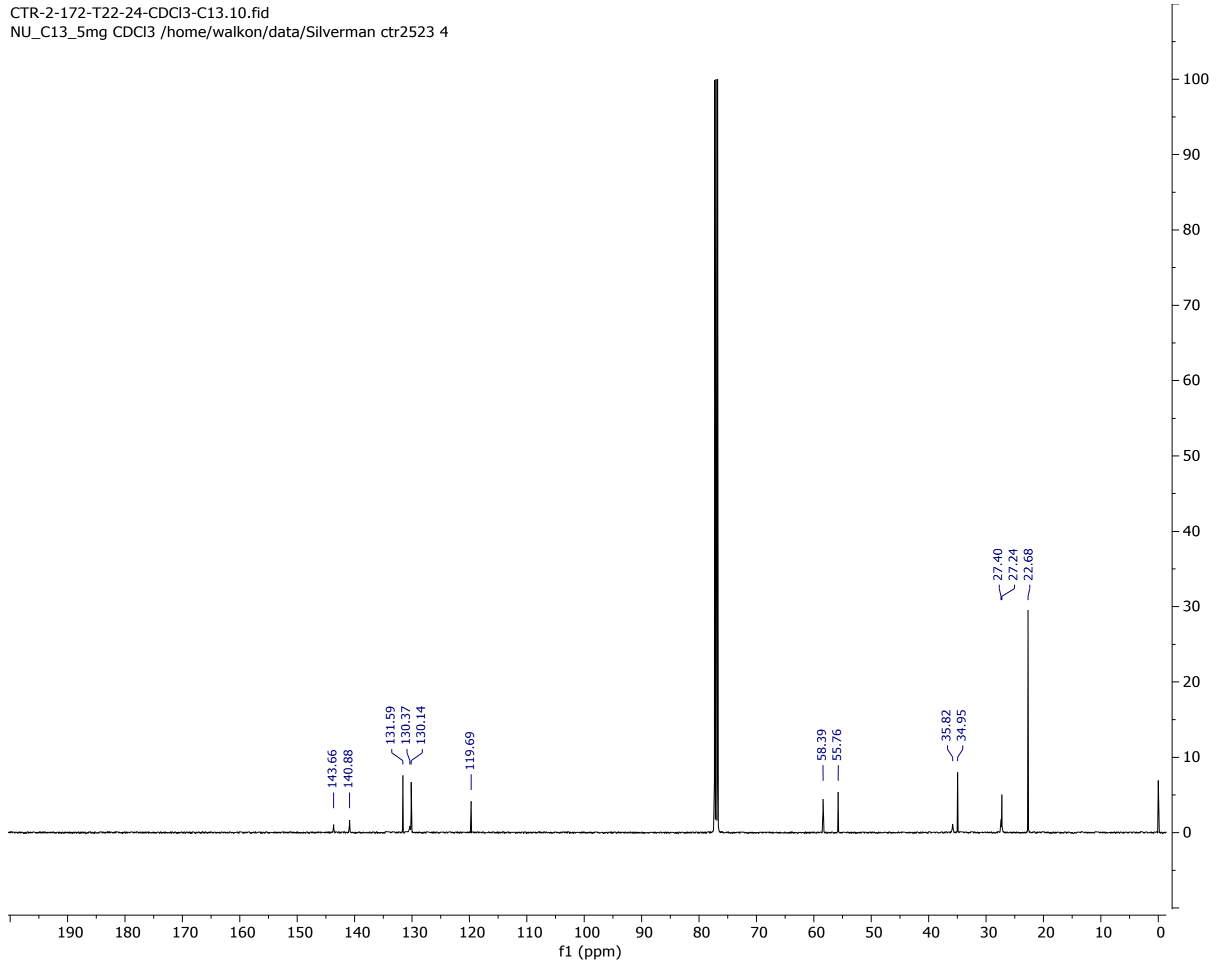

Figure S10. ^{13}C NMR for **(*S*)-N-((*R*)-3-bromo-6,7,8,9-tetrahydro-5H-benzo[7]annulen-5-yl)-2-methylpropane-2-sulfinamide (CTR-2-172-T22-24).**

N-(7-((*S*)-9-(((*S*)-tert-butylsulfinyl)amino)-6,7,8,9-tetrahydro-5H-benzo[7]annulen-2-yl)-4-methylquinolin-2-yl)acetamide (CTR-3-87E). In a 20 mL glass vial equipped with stir bar and septum cap was added XPhos-Pd-G3 (1.5 mg, 1.8 µmol), XPhos (1.7 mg, 3.6 µmol), tetrahydroxydiboron (47.9 mg, 0.53 mmol), and KOAc (52.4 mg, 0.53 mmol). EtOH (0.1 vol.) was charged followed by the addition of the **CTR-2-172-T17-19** (61.3 mg, 0.18 mmol). The reaction mixture was sealed under argon and heated to 80 °C for 2

hours. The reaction was then charged with 3 equivalents of degassed 1.8 M aqueous K_2CO_3 (0.3 mL, 0.53 mmol) followed by N-(7-bromo-4-methylquinolin-2-yl)acetamide (44.7 mg, 0.16 mmol.[2] The reaction mixture was flushed with argon and heated to 80 °C overnight. The reaction mixture was cooled to room temperature, diluted with water (5-10 mL) and extracted with EA (3 × 10 mL). The combined organics were dried over anhydrous Na_2SO_4, and concentrated under a nitrogen stream. The crude product was purified by normal phase flash column chromatography (12 g RediSep Gold® Silica Gel Disposable Flash Columns), eluting with a gradient of 100% CH_2Cl_2 to 100% EA, affording 43.7 mg of **CTR-3-87E** as a colorless residue in 62% yield. This material was used without further purification. ^{1}H NMR (500 MHz, $CDCl_3$) δ 9.92 (s, 1H), 8.30 (s, 1H), 7.99 (dd, *J* = 5.2, 3.4 Hz, 2H), 7.78 (dd, *J* = 8.7, 1.7 Hz, 1H), 7.65 (d, *J* = 2.0 Hz, 1H), 7.54 – 7.49 (m, 1H), 7.22 (d, *J* = 7.7 Hz, 1H), 4.80 – 4.65 (m, 1H), 3.51 (d, *J* = 3.1 Hz, 1H), 3.16 – 3.05 (m, 1H), 2.75 (s, 3H), 2.33 (s, 3H), 2.30 – 2.17 (m, 2H), 2.11 – 1.67 (m, 4H), 1.67 – 1.46 (m, 1H), 1.28 (s, 9H). ^{13}C NMR (126 MHz, $CDCl_3$) δ 171.17, 150.51, 143.23, 142.57, 137.37, 131.22, 130.96, 127.68, 126.67, 126.14, 125.28, 124.80, 124.73, 114.03, 60.41, 59.17, 58.99, 56.00, 55.86, 35.38, 31.83, 29.32, 28.10, 27.93, 25.83, 25.55, 25.04, 22.82, 21.06, 19.50, 14.21, 1.0.

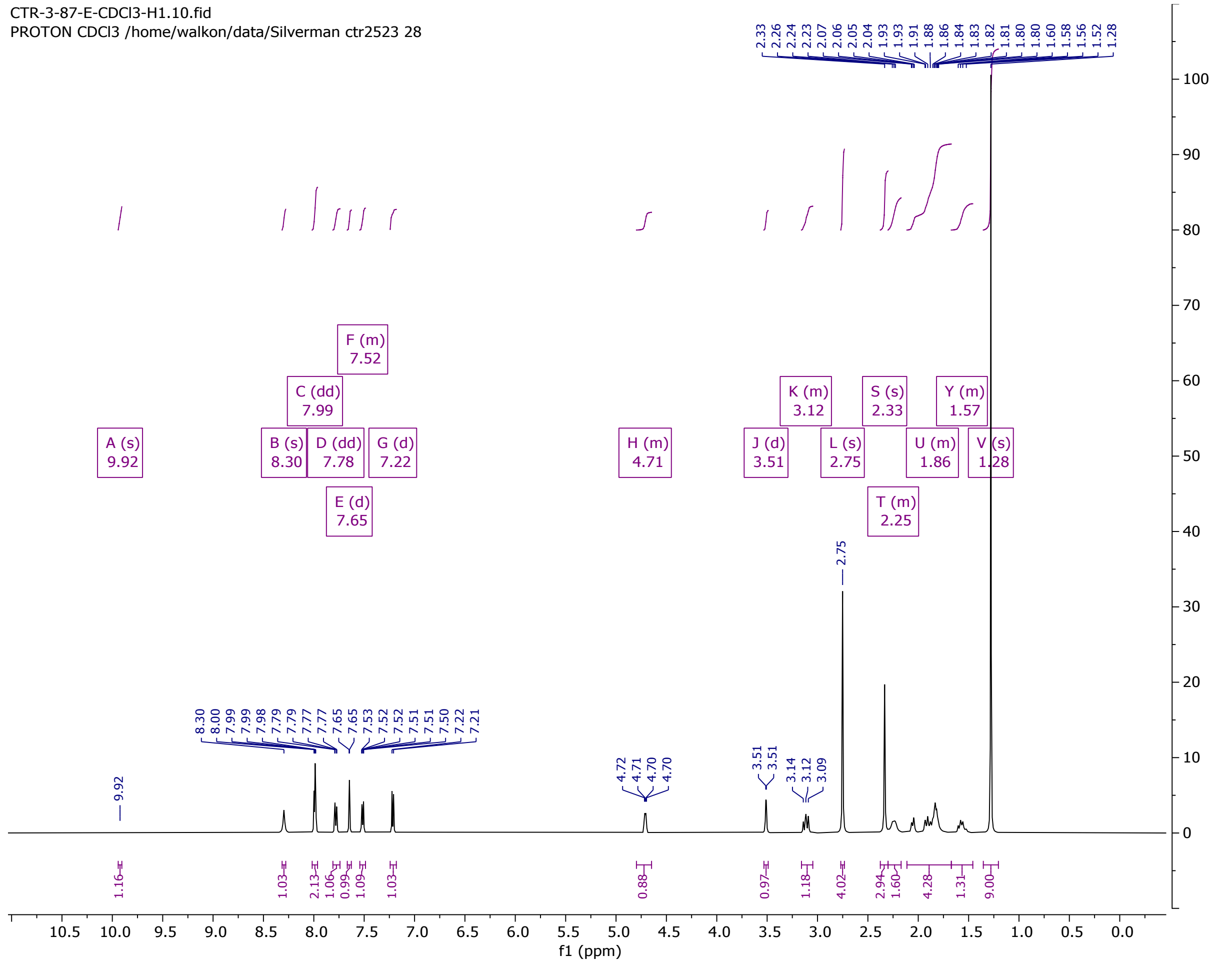

Figure S11. ^{1}H NMR for **N-(7-((*S*)-9-(((*S*)-tert-butylsulfinyl)amino)-6,7,8,9-tetrahydro-5H-benzo[7]annulen-2-yl)-4-methylquinolin-2-yl)acetamide (CTR-3-87E).**

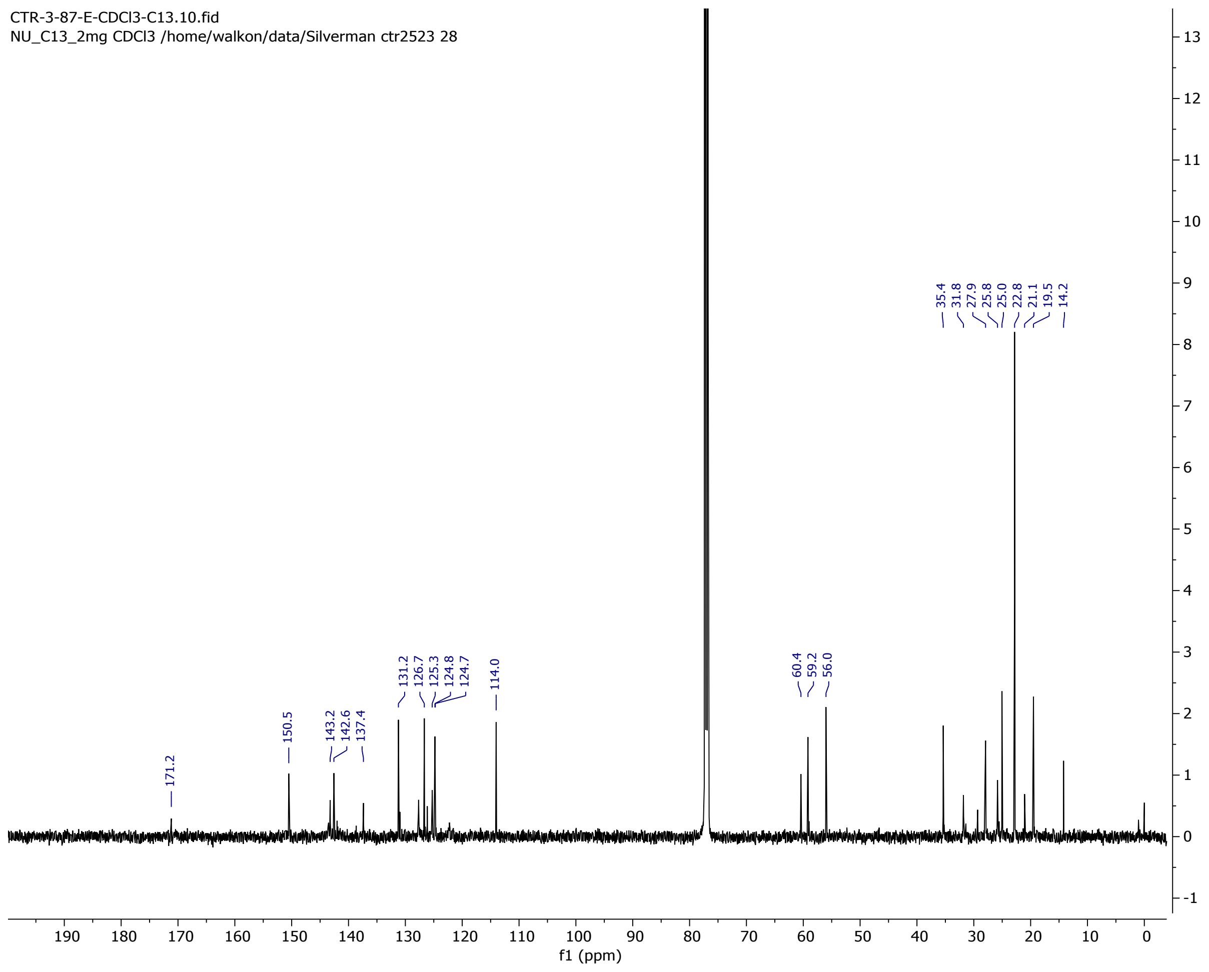

Figure S12. ^{13}C NMR for **N-(7-((*S*)-9-(((*S*)-tert-butylsulfinyl)amino)-6,7,8,9-tetrahydro-5H-benzo[7]annulen-2-yl)-4-methylquinolin-2-yl)acetamide (CTR-3-87E).**

(*S*)-7-(9-amino-6,7,8,9-tetrahydro-5H-benzo[7]annulen-2-yl)-4-methylquinolin-2-amine Dihydrochloride (6). To **CTR-3-87E** (39.9 mg, 0.09 mmol) concentrated to a residue in a 2 mL microwave vial was added 3.0 M methanolic HCl (1.5 mL, 4.5 mmol). The reaction vial was capped and heated for two cycles of 75 °C for 60 min in a

microwave reactor. After cooling, the reaction was concentrated under a stream of nitrogen. The residue was taken up in minimal hot methanol and dripped into 100-200 mL of anhydrous ether at room temperature to precipitate the title compound which was subsequently collected via vacuum filtration and washed with excess ether to afford 32.3 mg of **6** as a beige solid in 96% yield. ^{1}H NMR (500 MHz, CD_3OD) δ 8.02 (d, *J* = 8.5 Hz, 1H), 7.86 (d, *J* = 1.8 Hz, 1H), 7.78 (dd, *J* = 8.5, 1.8 Hz, 1H), 7.58 (dd, *J* = 7.8, 1.9 Hz, 1H), 7.52 (d, *J* = 1.9 Hz, 1H), 7.31 (d, *J* = 7.8 Hz, 1H), 6.83 (d, *J* = 1.3 Hz, 1H), 4.62 (d, *J* = 9.9 Hz, 1H), 2.94 – 2.81 (m, 2H), 2.63 (d, *J* = 1.1 Hz, 3H), 2.10 – 2.02 (m, 1H), 2.01 – 1.81 (m, 2H), 1.73 (qd, *J* = 10.6, 5.5 Hz, 1H), 1.39 – 1.33 (m, 1H). ^{13}C NMR (126 MHz, CD_3OD) δ 154.2, 153.6, 144.7, 142.1, 138.3, 137.1, 136.2, 130.9, 126.7, 125.9, 124.0, 120.8, 114.7, 111.9, 53.9, 34.4, 32.8, 28.0, 26.6, 18.0. HRMS: calcd. for $C_{21}H_{24}N_3^+$ 318.1965, found 318.1962.

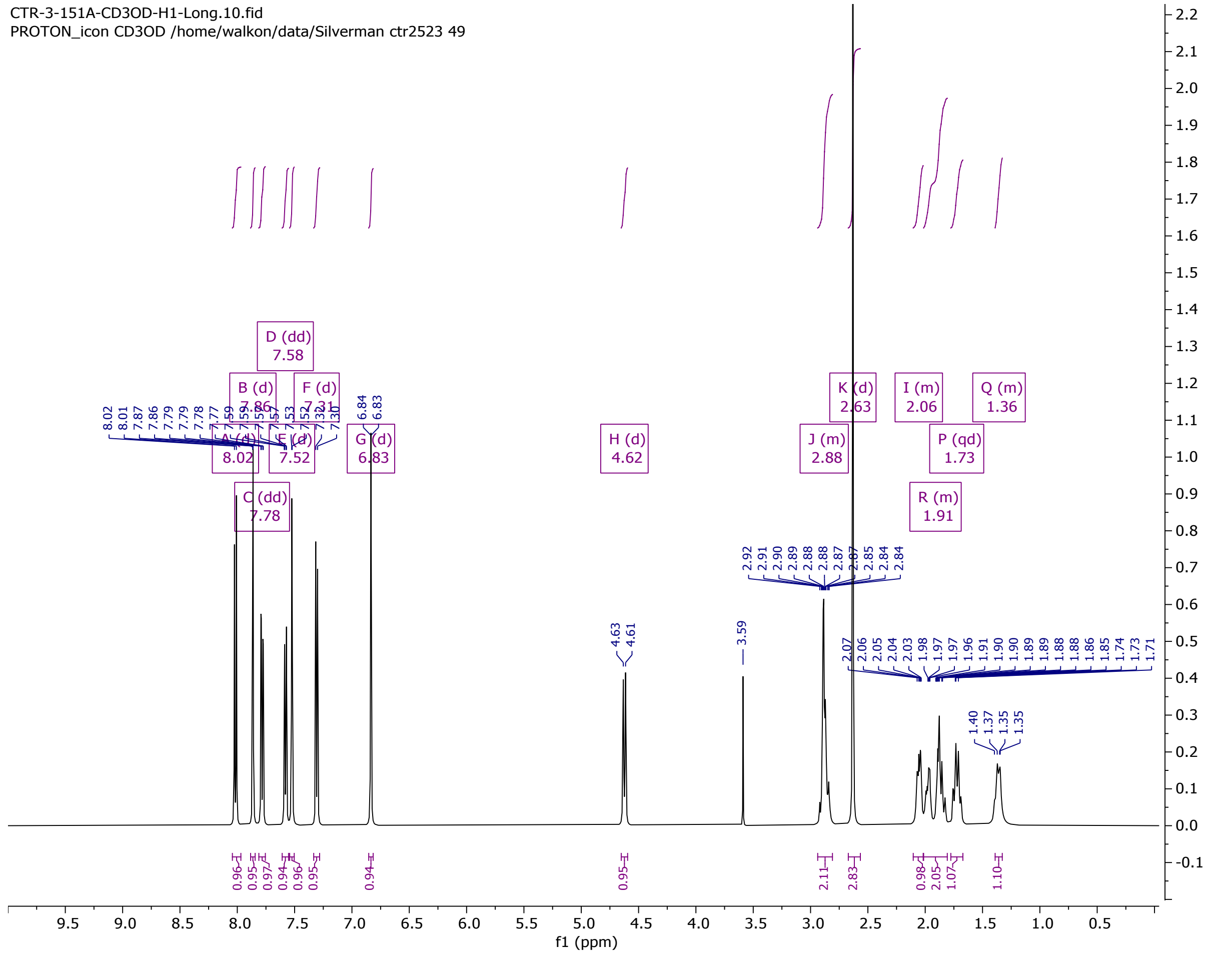

Figure S13. ^{1}H NMR for **(*S*)-7-(9-amino-6,7,8,9-tetrahydro-5H-benzo[7]annulen-2-yl)-4-methylquinolin-2-amine Dihydrochloride (5).**

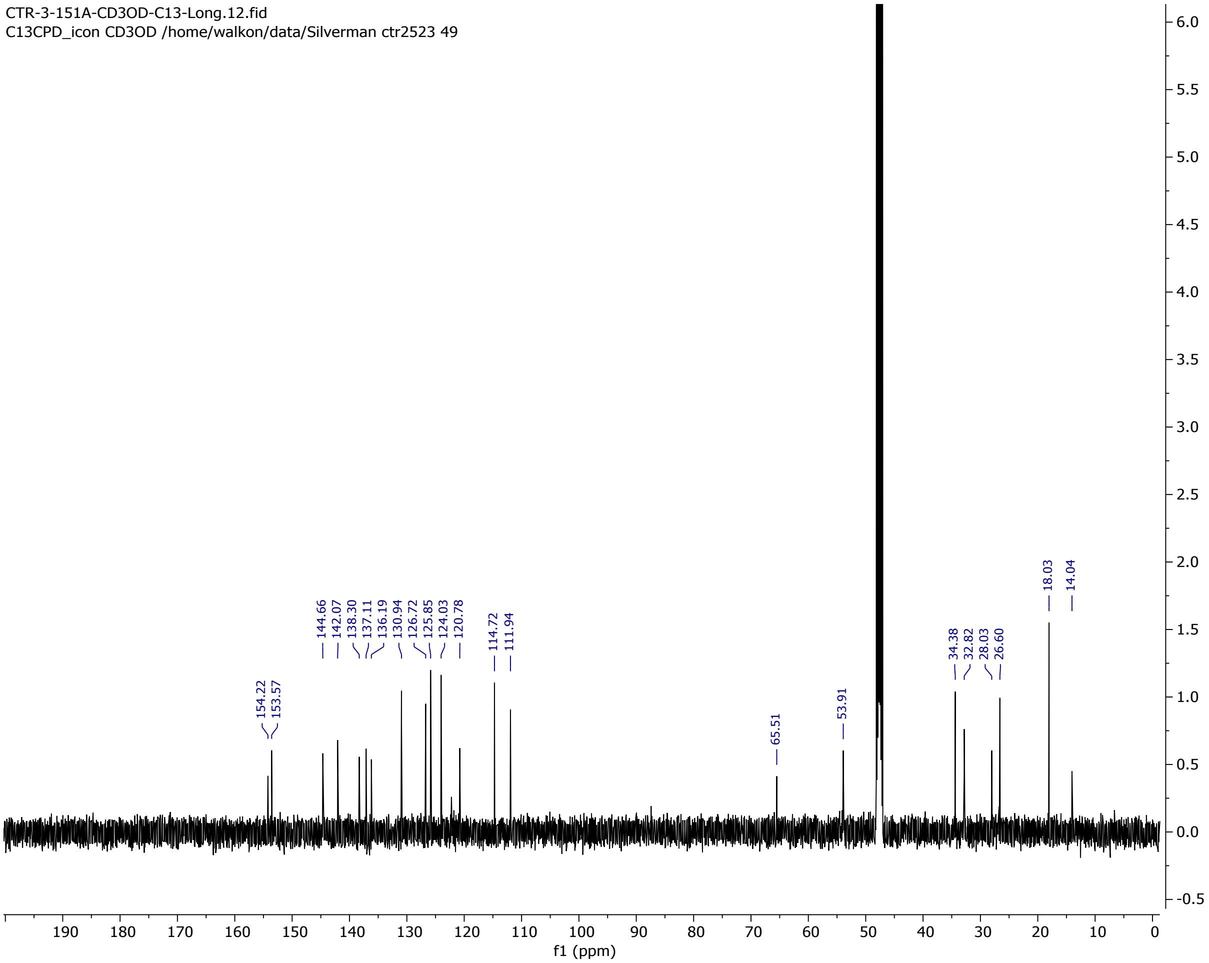

Figure S14. ^{13}C NMR for **(*S*)-7-(9-amino-6,7,8,9-tetrahydro-5H-benzo[7]annulen-2-yl)-4-methylquinolin-2-amine Dihydrochloride (5).**

N-(7-((*R*)-9-(((*S*)-tert-butylsulfinyl)amino)-6,7,8,9-tetrahydro-5H-benzo[7]annulen-2-yl)-4-methylquinolin-2-yl)acetamide (CTR-3-59C). In an 20 mL glass vial equipped with stir bar and septum cap was added XPhos-Pd-G3 (3.8 mg, 4.5 μmol), XPhos (3.0 mg, 6.3 μmol), tetrahydroxydiboron (89.1 mg, 0.99 mmol), and KOAc (103 mg, 1.0 mmol). EtOH (0.1 vol.) was charged followed by the addition of the **CTR-2-172-T22-24** (91.3 mg, 0.27 mmol). The reaction mixture was sealed under argon and heated to 80 °C

for 2 hours. The reaction was then charged with 3 equivalents of degassed 1.8 M aqueous K_2CO_3 (0.45 mL, 0.81 mmol) followed by **N-(7-bromo-4-methylquinolin-2-yl)acetamide** (44.7 mg, 0.16 mmol.[2] The reaction mixture was flushed with argon and heated to 80 °C overnight. The reaction mixture was cooled to room temperature, diluted with water (5-10 mL) and extracted with EA (3 × 10 mL). The combined organics were dried over anhydrous Na_2SO_4 and concentrated under a nitrogen stream. The crude product was purified normal phase flash column (12 g RediSep Gold® Silica Gel Disposable Flash Columns) chromatography, eluting with a gradient of 100% CH_2Cl_2 to 100% EA to afford 86.8 mg of **CTR-3-59** as a yellow glass in 70% yield. This material was used without further purification. ^1H NMR (500 MHz, $CDCl_3$) δ 9.91 (s, 1H), 8.33 (s, 1H), 8.10 – 7.95 (m, 2H), 7.81 (d, *J* = 8.6 Hz, 1H), 7.72 (s, 1H), 7.54 (dt, *J* = 7.7, 1.8 Hz, 1H), 7.24 (d, *J* = 7.7 Hz, 1H), 4.76 (dt, *J* = 5.7, 2.9 Hz, 1H), 3.44 (d, *J* = 3.7 Hz, 1H), 3.05 (t, *J* = 12.1 Hz, 1H), 2.82 (dd, *J* = 11.2, 6.2 Hz, 1H), 2.79 (s, 3H), 2.36 (s, 3H), 2.16 – 1.85 (m, 4H), 1.84 – 1.58 (m, 2H), 1.26 (d, *J* = 1.3 Hz, 9H). ^{13}C NMR (126 MHz, $CDCl_3$) δ 171.2, 150.5, 142.8, 137.0, 131.0, 126.4, 125.5, 124.7, 124.6, 114.0, 60.4, 59.2, 55.7, 35.3, 29.3, 27.7, 27.1, 25.0, 22.8, 21.1, 19.6, 14.2, 1.0.

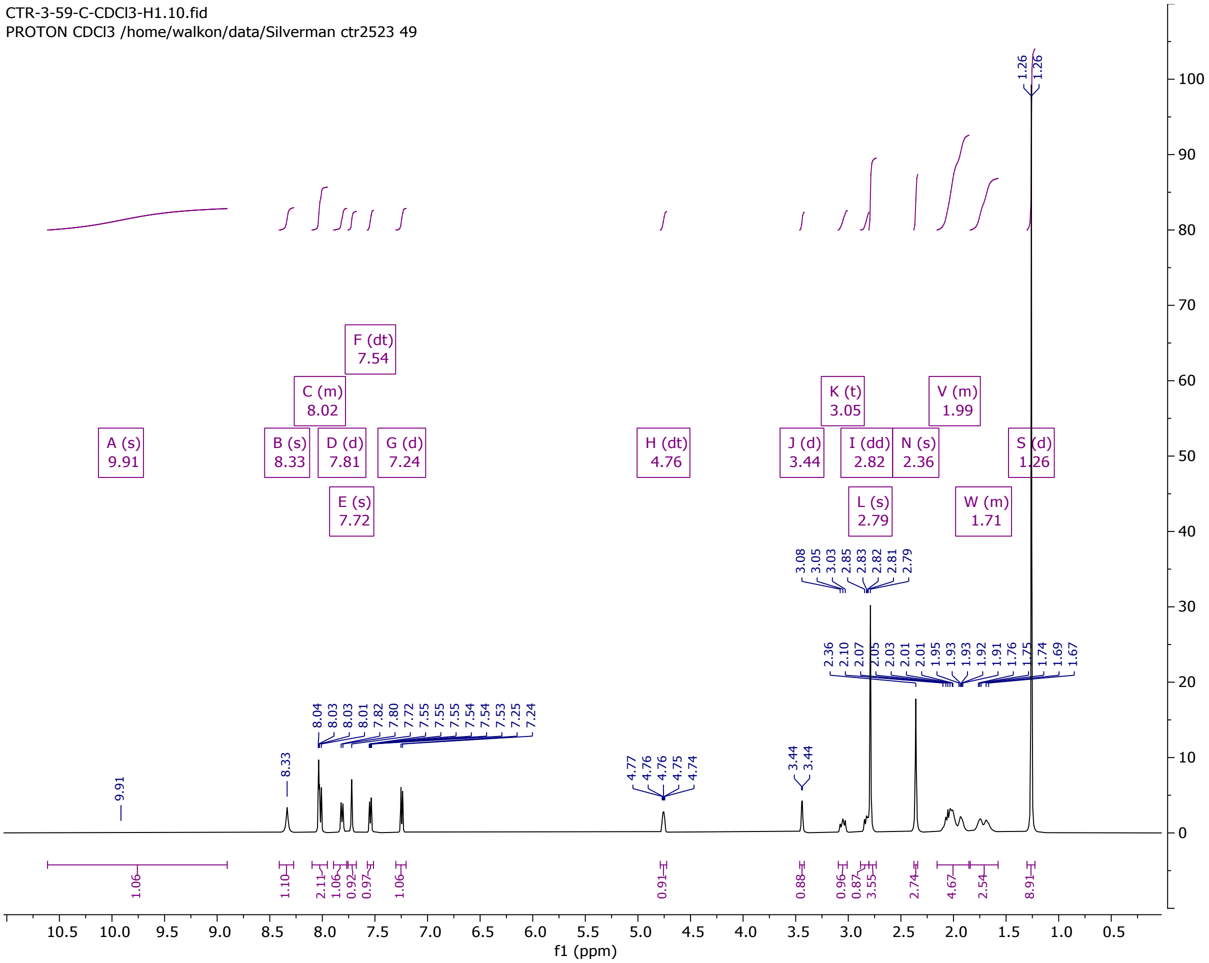

Figure S15. ^{1}H NMR for **N-(7-((*R*)-9-(((*S*)-tert-butylsulfinyl)amino)-6,7,8,9-tetrahydro-5H-benzo[7]annulen-2-yl)-4-methylquinolin-2-yl)acetamide (CTR-3-59-C).**

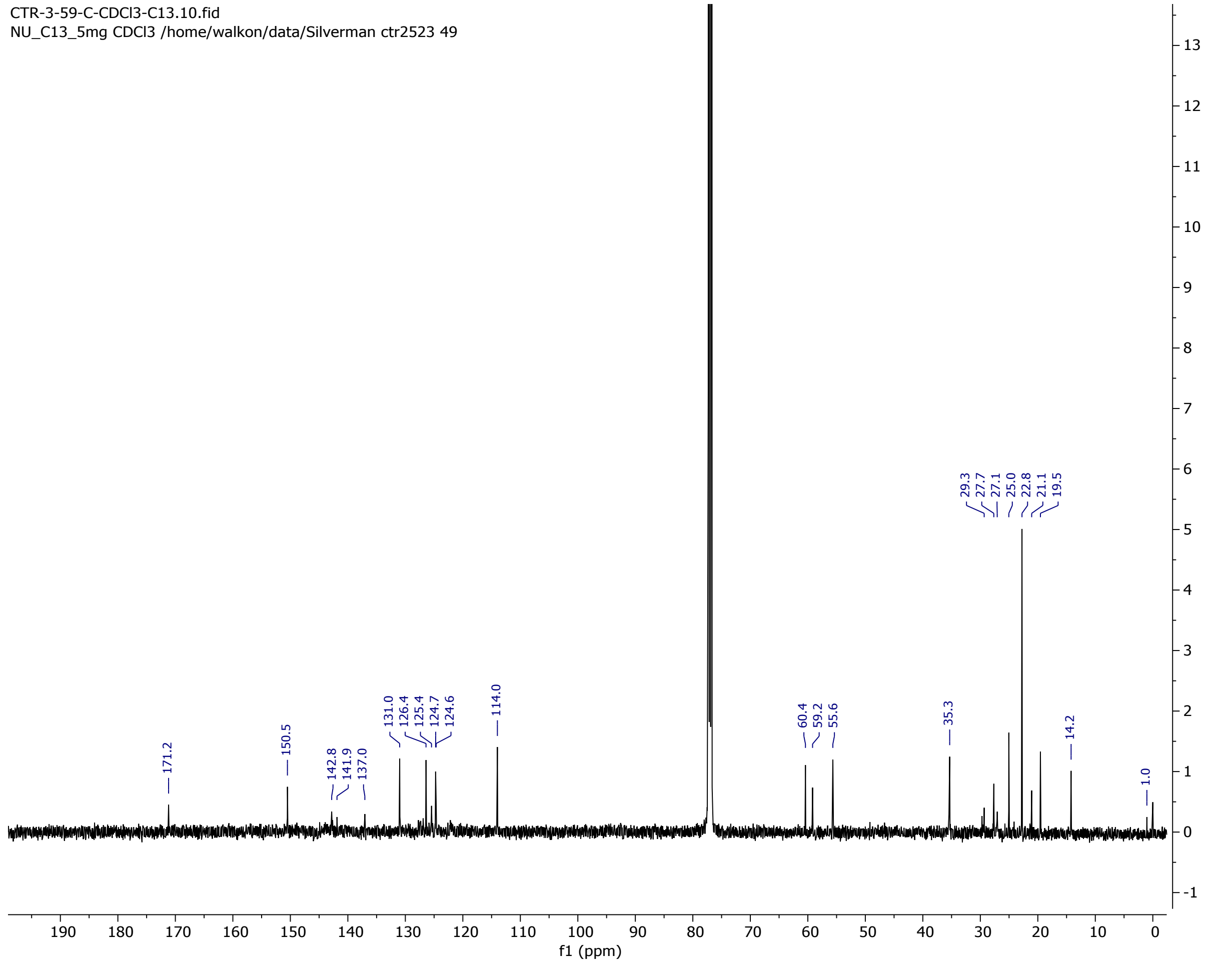

Figure S16. ^{13}C NMR for **N-(7-((*R*)-9-(((*S*)-tert-butylsulfinyl)amino)-6,7,8,9-tetrahydro-5H-benzo[7]annulen-2-yl)-4-methylquinolin-2-yl)acetamide (CTR-3-59-C).**

(*R*)-7-(9-amino-6,7,8,9-tetrahydro-5H-benzo[7]annulen-2-yl)-4-methylquinolin-2-amine Dihydrochloride (CTR-3-148). To **CTR-3-59C** (81.1 mg, 0.15 mmol) concentrated to a residue in a 2 mL microwave vial was added 3.0 M methanolic HCl (1.5 mL, 4.5 mmol). The reaction vial was capped and heated for two cycles of 75 °C for 60 min in a microwave reactor. After cooling, the reaction was concentrated under a

stream of nitrogen. The residue was taken up in minimal hot methanol and dripped into 100-200 mL of anhydrous ether at room temperature to precipitate the title compound which was subsequently collected via vacuum filtration and washed with excess ether to afford 55.2 mg of **CTR-3-148** as a white solid in 81% yield. MP = 268.4 °C (browned), 272.7 °C (softens), 275.3-277.0 °C (melt). ^{1}H NMR (500 MHz, CD_3OD) δ 8.02 (d, *J* = 8.6 Hz, 1H), 7.86 (d, *J* = 1.7 Hz, 1H), 7.79 (dd, *J* = 8.5, 1.8 Hz, 1H), 7.58 (dd, *J* = 7.8, 1.9 Hz, 1H), 7.52 (d, *J* = 1.8 Hz, 1H), 7.31 (d, *J* = 7.8 Hz, 1H), 6.84 (d, *J* = 1.2 Hz, 1H), 4.66 – 4.60 (m, 1H), 2.95 – 2.79 (m, 2H), 2.63 (d, *J* = 1.1 Hz, 3H), 2.02 (dddd, *J* = 40.0, 8.7, 5.2, 2.8 Hz, 2H), 1.87 (dddd, *J* = 20.6, 11.1, 5.3, 2.6 Hz, 2H), 1.73 (dtd, *J* = 13.5, 10.5, 2.9 Hz, 1H), 1.41 – 1.31 (m, 1H). ^{13}C NMR (126 MHz, CD_3OD) δ 154.2, 153.6, 144.7, 142.1, 138.3, 137.1, 136.2, 131.0, 126.7, 125.9, 124.1, 120.8, 114.7, 111.9, 53.9, 34.4, 32.8, 28.0, 26.6, 18.0. (ether peaks visible). HRMS: calcd. for $C_{21}H_{24}N_3^+$ 318.1965, found 318.1957.

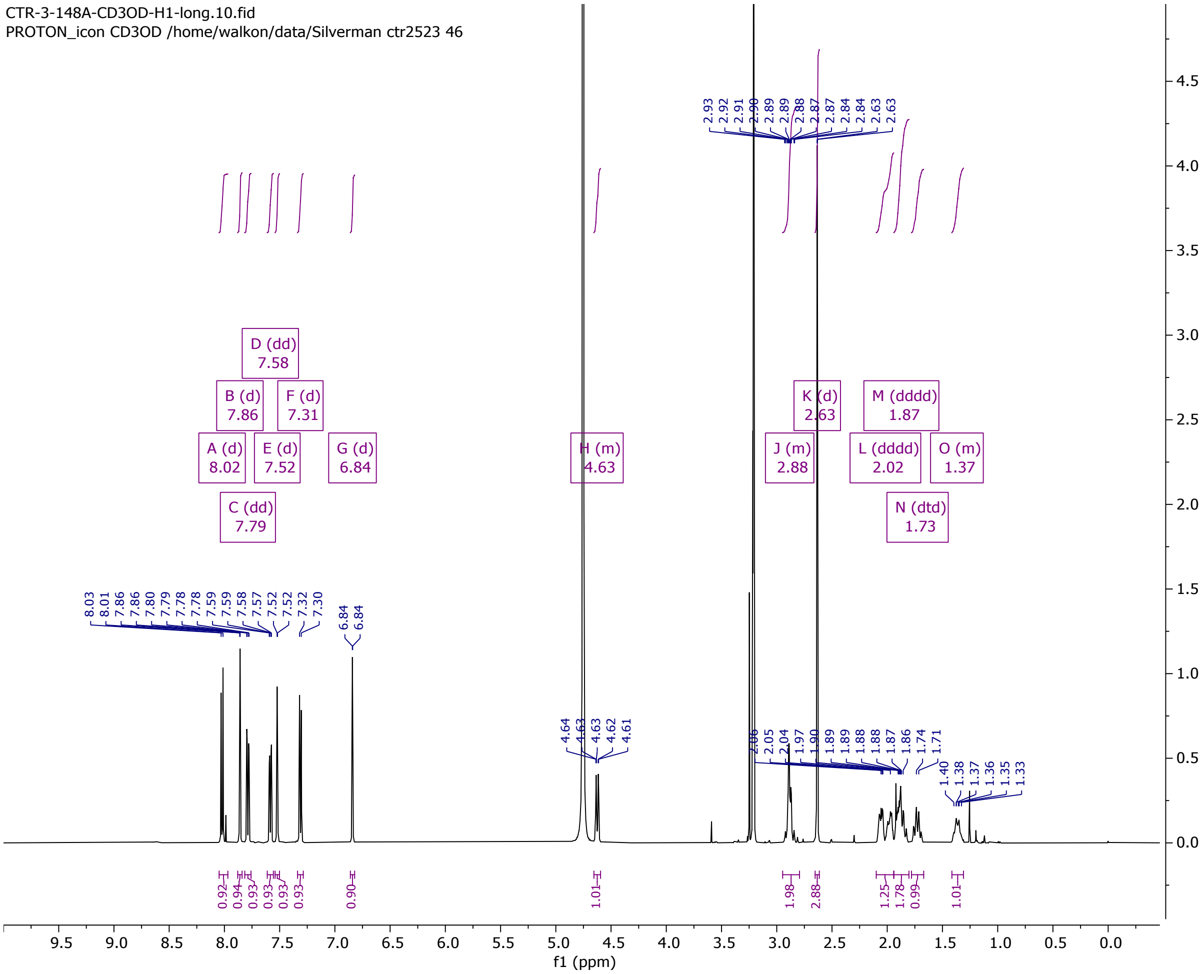

Figure S17. ^{1}H NMR for **(*R*)-7-(9-amino-6,7,8,9-tetrahydro-5H-benzo[7]annulen-2-yl)-4-methylquinolin-2-amine Dihydrochloride (CTR-3-148).**

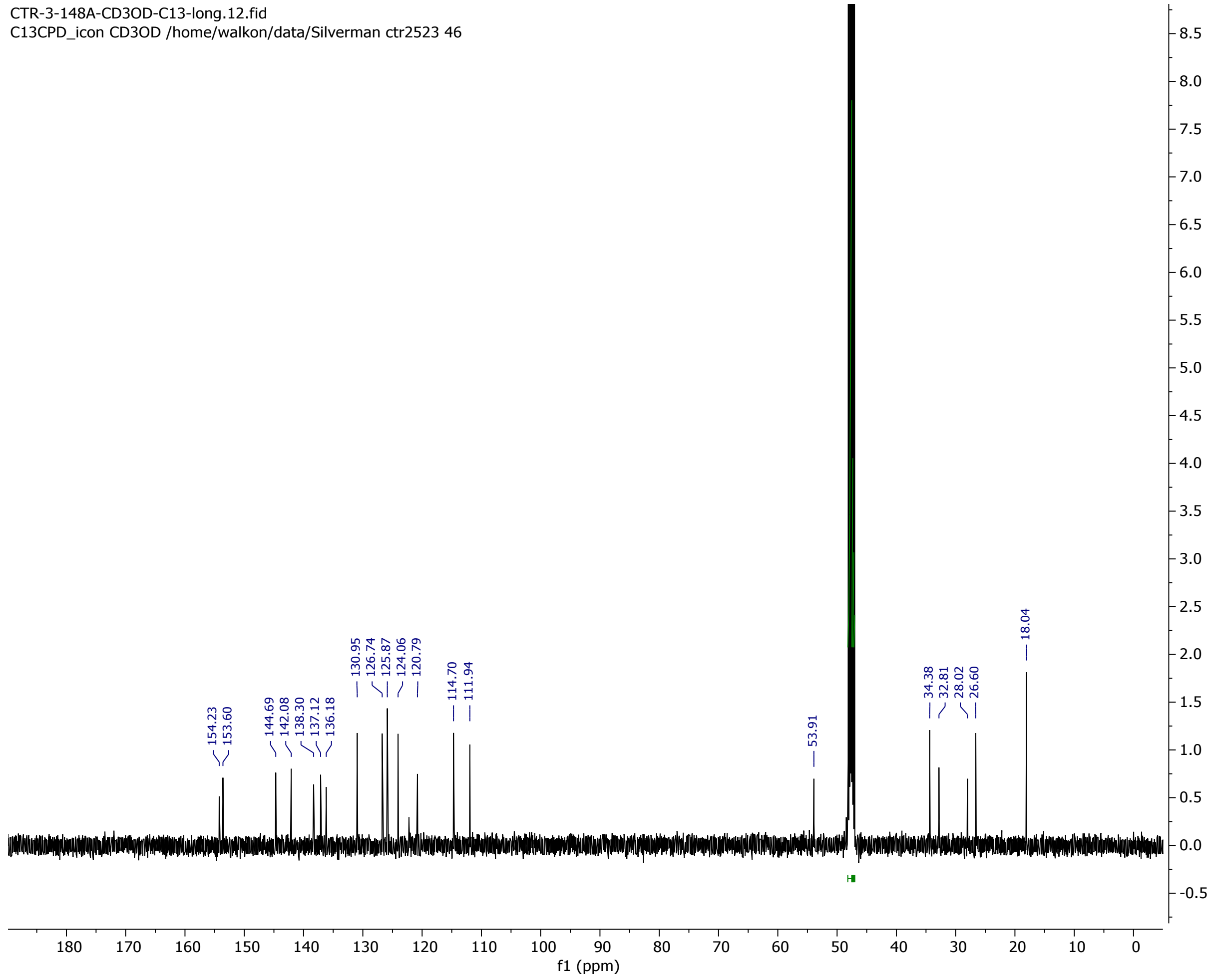

Figure S18. ^{13}C NMR for **(*R*)-7-(9-amino-6,7,8,9-tetrahydro-5H-benzo[7]annulen-2-yl)-4-methylquinolin-2-amine Dihydrochloride (CTR-3-148).**

References

[1] Cinelli, M.A.; Reidl, C.T.; Li, H.; Chreifi, G.; Poulos, T.L.; Silverman, R.B. First Contact: 7-Phenyl-2-Aminoquinolines, Potent and Selective Neuronal Nitric Oxide Synthase Inhibitors That Target an Isoform-Specific Aspartate. **2020**, *J. Med. Chem.* 63, 4528-4554.

[2] Vasu, D.; Reidl, C.T.; Wang, E.; Yang, S.; Silverman, R.B. Improved synthesis and anticancer activity of a potent neuronal nitric oxide synthase inhibitor **2023,** *Bioorganic & Bioorg. Med. Chem. Lett.* 90, 129329.
